# Transient Response and Ionic Dynamics in Organic Electrochemical Transistors

**DOI:** 10.1007/s40820-024-01452-y

**Published:** 2024-07-02

**Authors:** Chao Zhao, Jintao Yang, Wei Ma

**Affiliations:** State Key Laboratory for Mechanical Behavior of Materials, Xi’an Jiaotong University, Xi′an, 710049 People’s Republic of China

**Keywords:** Organic electrochemical transistors, Transient response, Ion dynamics, Electronic dynamics, Volatility and non-volatility

## Abstract

The rapid development of organic electrochemical transistors (OECTs) has ushered in a new era in organic electronics, distinguishing itself through its application in a variety of domains, from high-speed logic circuits to sensitive biosensors, and neuromorphic devices like artificial synapses and organic electrochemical random-access memories. Despite recent strides in enhancing OECT performance, driven by the demand for superior transient response capabilities, a comprehensive understanding of the complex interplay between charge and ion transport, alongside electron–ion interactions, as well as the optimization strategies, remains elusive. This review aims to bridge this gap by providing a systematic overview on the fundamental working principles of OECT transient responses, emphasizing advancements in device physics and optimization approaches. We review the critical aspect of transient ion dynamics in both volatile and non-volatile applications, as well as the impact of materials, morphology, device structure strategies on optimizing transient responses. This paper not only offers a detailed overview of the current state of the art, but also identifies promising avenues for future research, aiming to drive future performance advancements in diversified applications.

## Highlights

- Transient response plays a crucial role as a performance indicator for organic electrochemical transistors (OECTs), particularly in their application in high-speed logic circuits and neuromorphic computing systems.
- This review presents a systematic overview on the fundamental principles underlying OECT transient responses, emphasizing the essential roles of transient electron and ion dynamics, as well as structural evolution, in both volatile and non-volatile behaviors.
- We also discuss the materials, morphology, device structure strategies on optimizing transient responses.

## Introduction

Organic electrochemical transistors (OECTs) have emerged as a highly promising technological innovation in diverse domains, notably in sensing and neuromorphic electronics [1–11]. The ionic gating mechanism endows OECTs bulk doping with exceptional transconductance, ensuring high sensitivity and the ability to operate at low voltages [12]. The OECT is also cost-effective production, mechanical flexibility, and biocompatibility [13–17]. Leveraging a unique mechanism, OECTs showcase significant potential across a diverse array of applications. These range from logic circuits and biosensors to bio-inspired neuromorphic devices such as artificial synapses and organic electrochemical random-access memories (ECRAMs) [2,4,11,18–21]. Although inspired by biological processes, the application of OECTs extends beyond the bioelectronics domain. In the realm of logic circuits, OECTs are distinguished by their signal processing capabilities. Recent advancements, especially in vertical and internally gated device architectures, have facilitated the development of OECTs with rapid temporal dynamics [4,22–24]. This progression spans from sub-millisecond to sub-microsecond time scales. In the field of memory devices, OECTs employ ion dynamics to facilitate non-volatile memory functions, positioning OECTs at the forefront of mimicking both short-term and long-term plasticity (STP, LTP), spike-timing-dependent plasticity (STDP) functions, and even ECRAMs [4,25,26]. Through careful manipulation of ion transport dynamics, studies have demonstrated ECRAMs to execute rapid write pulses (20 ns) for programming synaptic weights, with impressive state retention capabilities that achieve more than 10-bit states [4,20]. In recent years, the high signal amplification capabilities of OECTs have showcased superior performance in multimodal sensing, including molecular detection at femtomolar levels, or even at the single-molecule level, and high-sensitivity light sensing based on OECTs, integrating photonic neuromorphic features.

To meet the varied application requirements for OECTs, particularly in terms of their transient response performance, which primarily evaluates how the OECT response to an input signal evolves until a new equilibrium state is achieved, it is necessary to understand the physics of transient response and explore methods for optimization. Compared to field-effect transistors, the operation of OECTs involves a complex interplay of charge and ion transport, along with electron–ion coupling interactions [27–31]. For instance, ions can transport within the channel and accumulate at the drain/source electrodes, leading to a nonlinear ion and charge distribution along the channel, making the understanding of ion transport processes, and their impact on the steady-state and transient responses of OECTs, significantly more complex. Key figures of merit for OECTs, such as transconductance, response speed, and non-volatility, are underlined by these dynamics [13,29]. Transconductance is primarily dictated by device geometry, charge mobility, and volumetric capacitance. Typically, charge mobility is much higher than ion transport, the latter varying greatly across different materials, ranging from 10^–10^ to 10^–3^ cm^2^ V^−1^ s^−1^ [32,33]. Consequently, the transient response speed is often determined by ion transport. In addition, non-volatility is also strongly influenced by ion dynamics—specifically, whether ions diffuse back once the gate voltage is removed [4,20,34]. In OECTs, ion dynamics are affected by many factors such as materials and morphology [35]. For example, in aqueous electrolytes, ions move faster within organic mixed ionic–electronic conductors (OMIECs) featuring alkoxy side chains. Ion also tends to transport in the amorphous phase than crystalline phase due to its larger transport energy barriers [9,36,37]. Additionally, gate voltage affects ion doping levels, with increased gate voltage encouraging ion doping into the crystalline regions of OMIECs, thus altering ion dynamics. Furthermore, under operational conditions, changes in ion and charge concentration can lead to variations in transport dynamics, such as mobility alterations with concentration [38]. Applying a gate voltage causes ions and water to inject into the channel, leading to swelling and changes in the microstructure, such as π–π stacking distances and polaron–bipolaron interaction-induced structural changes [39]. These structural changes, in turn, affect ion and charge transport. So far, a fundamental understanding of the correlation between dynamical microscopic structural changes and device performance in operational conditions is still in its nascent stages.

Given the complexity of these electron–ion coupled systems, fully understanding the scientific principles behind key physical processes, especially how they contribute to the transient response, remains challenging. Although there is some understanding of the physical principles and control methods regarding the transient response of OECTs, many aspects remain unclear and merit further investigation in the future. This review aims to delineate the burgeoning field of OECTs, focusing on principles of transient response behaviors, and strategies to manipulate ion dynamics. We embark on a detailed exploration of the working principles of OECTs, starting with a fundamental understanding of the electronic and ionic dynamics as laid out by the Bernards model, and progressing through recent advancements that shed light on the transient response. The review delves into the electron and ion dynamics, as well as structural evolution that OECTs undergo during operation, alongside the methods employed for their characterization (Sect. 2). As OECTs carve a niche for themselves across a wide range of applications—from volatile logic transistors and sensors to the non-volatile neuromorphic devices like artificial synapses and ECRAMs—we also review the understanding and manipulating ion dynamics for enhancing device functionality (Sects. 3 and 4). Specific attention is devoted to material selection, morphological adjustments, and the influence of device geometry on performance. Moreover, the review broadens the discourse to include the role of OECTs in applications beyond traditional electronics, such as photo-response, pressure sensing, and molecular detection, highlighting the interplay between ion dynamics and device functionality (Sect. 5). Finally, we investigate the future research and development of the physics and optimization of OECT transient response (Sect. 6).

## Working Principle of OECTs

### Basic Principles of OECTs

**Fig. 1:**
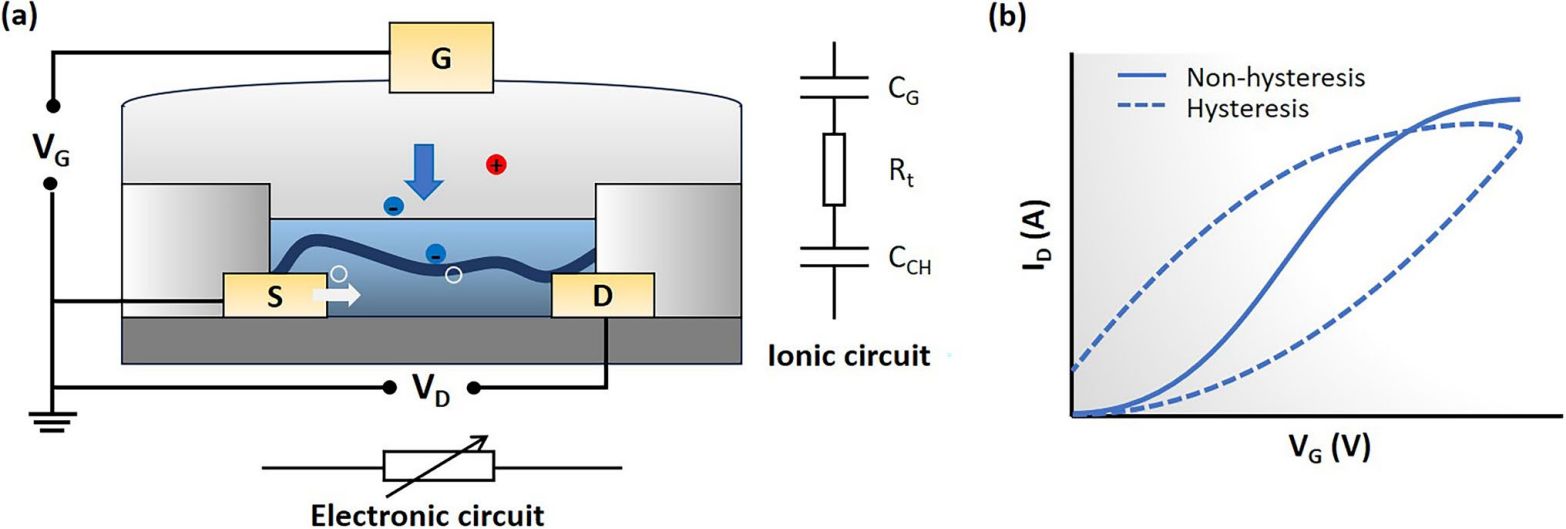
**a** Diagram illustrating the typical *p*-type OECT structure. Upon application of *V*_G_ (< 0 V), anions are injected into the channel, and are counterbalanced by the holes injected from source electrode, leading to the electrochemical doping of the channel. The ionic circuit and electronic circuit are also illustrated. **b** The transfer curve, where solid line represents non-hysteresis, and the dashed line represents the curve with hysteresis

The principles of OECTs operation have been widely described in the literatures [27]. The OECTs is a three-terminal that consists of two electrodes, i.e., the source and drain, connected by a semiconducting polymer, forming the channel. There is also an organic layer touching an electrolyte solution, with a gate electrode inside this solution. When we apply voltage to the gate, driven by the entropy of the mixings [28], it injects ion from the electrolyte into the channel, and gets compensated by the electron (*n*-type) or holes (*p*-type) injected from the source electrode [31] (see Fig. 1a). This can either add to (dope) or remove (dedope) charges in the semiconducting polymer, changing the conductivity and thus current between the source and drain [29].

OECTs can work in either depletion mode or accumulation mode. In depletion mode, the transistor starts on (because the channel is already doped) and turns off when the gate voltage is applied (which dedopes the channel). In accumulation mode, the device starts off (with the semiconducting polymer neutral) and turns on when the gate voltage causes ions to enter the polymer from the electrolyte. Upon the application of a gate voltage, ions are injected from the electrolyte into the OMIEC channel, balanced by charge injection from the source electrode. This increases the charge density in the polymer, enhancing its conductivity. Taking p-type OECTs as an example, this process is described by the reaction:

1 $${P}^{0}+{Q}^{-}-{e}^{-}\leftrightarrow {P}^{+}{Q}^{-}$$

where *P* is the polymer, *Q*^−^ is the anion, and *e*^−^ is an electron. During electrochemical doping of *P* by ${Q}^{-}$, an electron is removed from the polymer, resulting in the formation of doped ${P}^{+}$ which binds with the anion to maintain local neutrality. N-type doping is similar but uses cations. The system keeps a bulk neutrality by ensuring each ion that enters the polymer is matched with an electronic charge, creating a one-to-one match between ionic and electronic charges.

#### Bernards–Malliaras Model

An essential consideration in OECTs physics is described by the Bernards model [29]. It suggests that when ions enter the channel from the electrolyte, they do not chemically react with the polymer but change its conductivity by balancing out opposite charges. The model breaks the device into two parts: an ionic circuit (ions moving in the electrolyte and channel) and an electronic circuit (charge moving in the source, channel, and drain like a resistor). Therefore, the electronic circuit is treated as a resistor, in which electronic charge drifts under the influence of the local potential in a fashion identical to that of MOSFETs. The ions in the channel act more like a bulk capacitor, storing without reacting. This model implies a purely capacitive process, according to which ions injected in the channel do not exchange charge with the organic film but rather electrostatically compensate the presence of opposite charges. At steady state, the capacitor is fully charged (or discharged), and the gate current goes to zero. The Bernards model have achieved great success in fitting for the output characteristics of OECTs and allows quantitative analysis of the device electrical parameters. For *p*-type OECTs, it gives [29]:

2 $${I}_{CH}=\left\{\begin{array}{cc}\mu {C}^{*}\frac{Wd}{L}\left[{V}_{T}-{V}_{G}+\frac{1}{2}{V}_{D}\right]{V}_{D},& \text{ for }{V}_{D}>{V}_{G}-{V}_{T}\\ -\mu {C}^{*}\frac{Wd}{L}\frac{{\left[{V}_{G}-{V}_{T}\right]}^{2}}{2},& \text{ for }{V}_{D}<{V}_{G}-{V}_{T}\end{array}\right\}$$

Hence, the transconductance, defined as the derivative of channel current with respect to gate voltage (${g}_{\text{m}}=\frac{\partial {I}_{CH}}{\partial {V}_{G}}$), is given by:

3 $${g}_{\text{m}}=\left\{\begin{array}{cc}-\mu {C}^{*}\frac{Wd}{L}{V}_{D},& \text{ for }{V}_{D}>{V}_{G}-{V}_{T}\\ -\mu {C}^{*}\frac{Wd}{L}({V}_{G}-{V}_{T}),& \text{ for }{V}_{D}<{V}_{G}-{V}_{T}\end{array}\right\}$$

where *W*, *L* and *d* are the channel width, length and thickness, respectively; *μ* is the charge carrier mobility; C^*^ is the capacitance per unit volume of the channel [40]; and *V*_T_ is the threshold voltage. This fundamental equation bears resemblance to that used for field-effect transistors, with the notable difference being that *d*⋅*C*^∗^ substitutes the capacitance per unit area of the FET capacitor, underscoring the distinction between the bulk doping of OECTs and interface doping of other transistor types. The electrical behavior of OECTs parallels that of traditional transistors, with steady-state electrical characteristics depicted through transfer curves, as illustrated in Fig. 1b.

Based on the quasi-static approximation that assumes the charge distribution within the channel mirrors the steady-state solution for instant terminal voltages (*V*_S_​, *V*_D_​, and *V*_G_​), even when these voltages change over time, Bernards and Malliaras derived the OECT transient response formula. When *V*_DS_ voltages remain constant but the gate voltage fluctuates, the drain current’s time dependency is primarily influenced by the ionic RC circuit’s transient response, thus determining the voltage across the electrolyte, *V*_G,sol_​(t). Understanding *V*_G,sol_​(t) enables calculation of the ionic displacement current *i*_G_​(t) and the electronic transport current *i*_G_​(t). The displacement current, *i*_CH​_(t), linked to the doping or dedoping process in the OECTs channel, is defined as ${i}_{G}(t)={C}_{CH}\times \frac{d}{dt}{v}_{G,sol}(t)$, representing the current through the RC circuit. The electronic transport current, *i*_CH_​(t), represents the movement of electronic carriers between the source and drain​. The total drain current *I*_D_​(t) is a composite of the displacement and channel currents. Bernards derived the *I*_D_​(t) under a square gate voltage step: exponential dependency on time as ${i}_{D}(t)={I}_{SS}\left({V}_{G}\right)+\Delta {I}_{SS}\left[1-f\frac{{\tau }_{e}}{{\tau }_{i}}\right]\text{exp}\left(-\frac{t}{{\tau }_{i}}\right)$, where *I*_D_​(t) is the drain current at time *t*; *I*_SS_​(*V*_G_​) is the steady-state drain current for a given gate voltage; Δ*I*_SS_​ is the change between initial and final steady-state currents; *f* is a factor of weighting; *τ*_e​_ is the electronic transit time; and *τ*_i_​ is the ionic RC time constant [29].

**Fig. 2:**
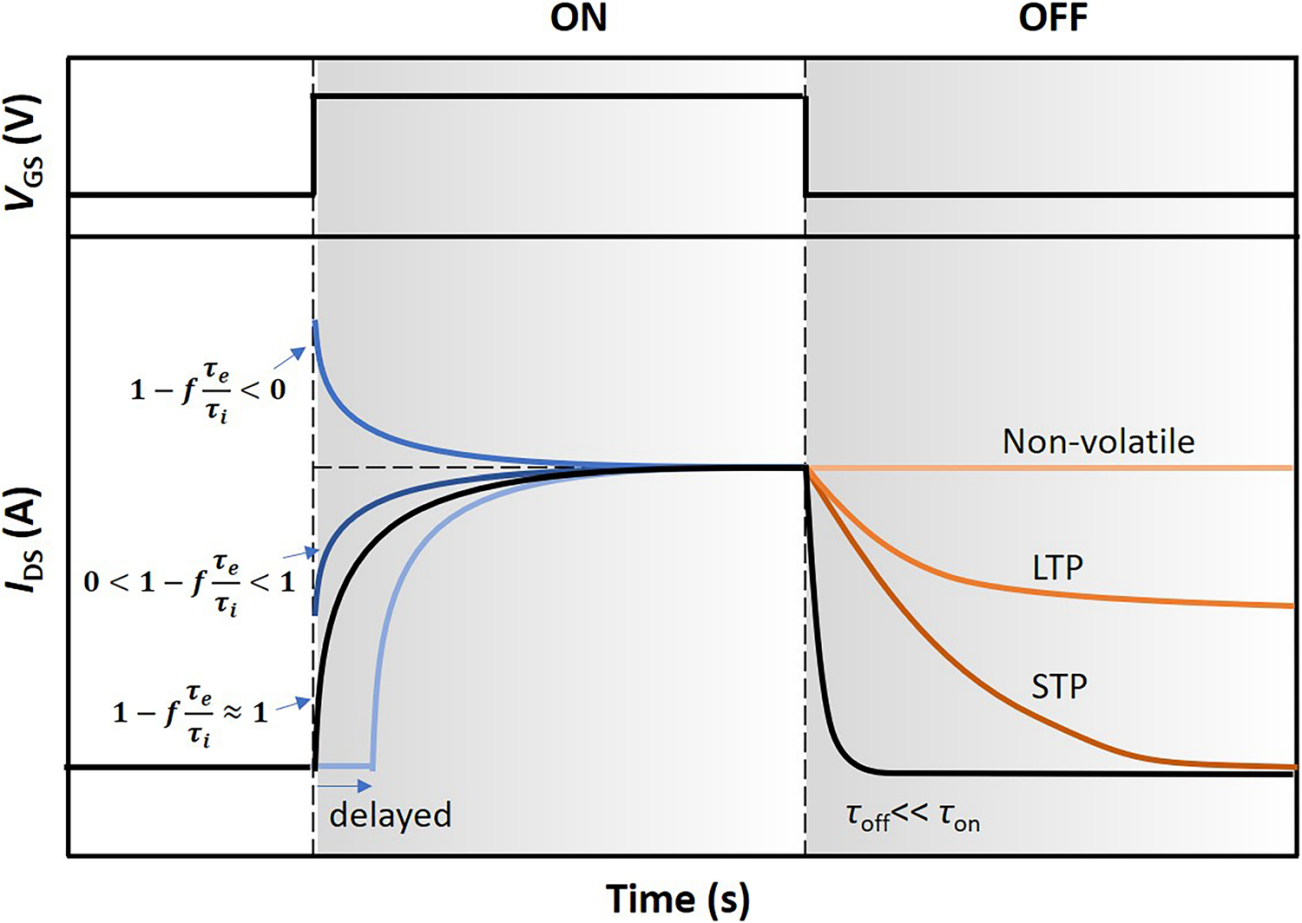
Summary of various transient response behaviors in OECTs. In the on-switching phase, typical behaviors include monotonic decay ($1-f\frac{{\tau }_{e}}{{\tau }_{i}}\approx 1$), sudden increase followed by monotonic decay ($0<1-f\frac{{\tau }_{e}}{{\tau }_{i}}<1$), spike-and-recovery ($1-f\frac{{\tau }_{e}}{{\tau }_{i}}<0$), and delayed curve. In the off-switching phase, typical transient behaviors include fast decay (*τ*_off_ <  < *τ*_on_), slow decay (STP), slow decay with certain memory (LTP), and no decay (non-volatile)

This model leads to two primary conclusions about OECTs transient responses: first, the transit time of an OECTs is fundamentally governed by the ionic doping process, specifically *τ*_i​_ in the exponential decay term, regardless of electronic transit times. Also, by precisely controlling *f*, *τ*_e_​, and *τ*_i_​, the time required for *I*_D_​ to reach its steady-state value *I*_SS_​ can be minimized over ionic transit limit, enhancing device performance (Fig. 2). The electronic transit time depends on the channel length and the mobility of charge carriers. On the other hand, the ionic RC time constant is determined by the channel’s total capacitance (*C* × *WdL*), and the series resistance within the ionic circuit, which strongly depends on ion mobility. These highlight the profound impact of the OECT’s geometry and materials on switching speeds. Devices with larger geometries may require longer pulse lengths to achieve high-resolution current responses, as suggested by previous studies by Bernards (2007), Faria (2017), Rivnay (2015), and Friedlein (2016) [29,41,42].

Analysis of the OECT’s transient behavior identifies two distinct regimes based on the above equation that describes OECTs transient behaviors. The first regime occurs when electronic transport is quicker than ionic charging, leading to a monotonic relaxation of the drain current from its initial to its final state. The second regime, observed when ionic charging outpaces electronic transport, features an initial spike in drain current before it exponentially settles to its final value. These regimes have been confirmed by various studies, underscoring the dynamic responses OECTs can exhibit [29,41,42].

**Fig. 3:**
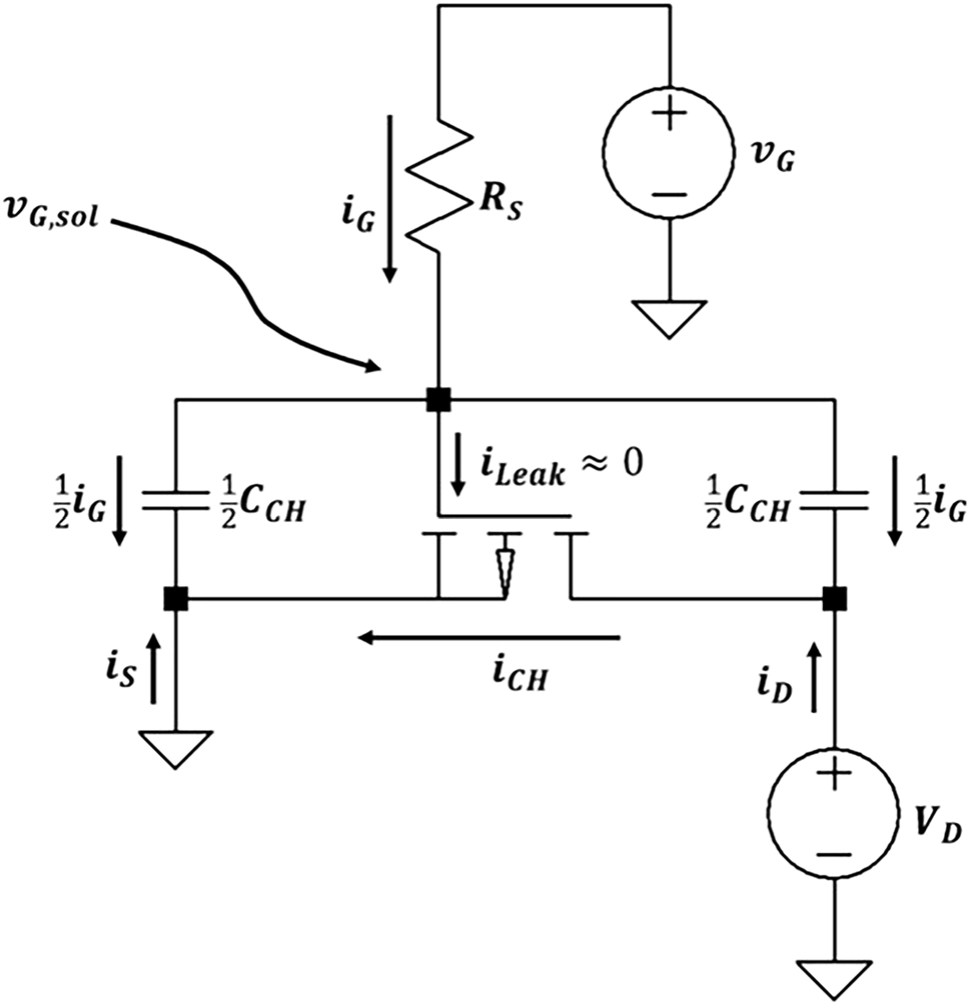
Circuit diagram of the equivalent model. Reproduced with permission [43]. Copyright 2016, Wiley–VCH

Another crucial parameter is the weighting factor *f*, pivotal in quantifying the displacement current’s role in the overall drain current. The treatment of *f* varies among models extending from the Bernards model, with investigations by Friedlein et al. [43], Faria et al. [42], and Tu et al. [44] delving into this aspect. Tu et al. adopted the Ward-Dutton partitioning scheme to define *f*, considering the distribution of mobile charges along the channel. This scheme deduces a voltage-dependent *f*, dividing the contribution of mobile charges at any position *x* between the drain and source currents. This approach has provided analytical expressions aligning qualitatively with experimental findings and offers a nuanced understanding of current transients in OECTs [44]. Friedlein proposed a simplification by setting *f* = 1/2, a constant value facilitating precise model predictions, as demonstrated in their experimental step response (Fig. 3). This approximation assumes that a change in channel current exactly half of the maximum gate current (0.5*I*_G,max​_ = Δ*I*_CH​_) will result in an immediate transition of the drain current to its final value, devoid of exponential decay [43]. Further, Faria studied the role of the *f* factor in accurately fitting and extracting impedances from OECTs, challenging the previous assumption that *f* = 1/2. By measuring gate and drain current responses to *V*_GS_ applications and fitting these responses with calculated impedances, they show that *f* varies depending on gate and drain voltages in a PEDOT:PSS OECT device. Notably, *f* approximates 0.5 when the gate voltage is positive and the drain voltage is zero, reflecting an indistinct pathway for charges between the source and drain. Deviations from 0.5 occur when the drain voltage is nonzero; *f* trends toward 1 with negative drain voltages, indicating a preferred pathway for positive charges to the drain, and toward 0 with positive drain voltages, indicating a non-preferred pathway. Therefore, *f* is contingent on the operational conditions of the OECTs, including the voltage settings and possibly the device’s geometric characteristics.

While the Bernards model and its associated equivalent circuit model effectively illustrate the monotonic and spike-and-recover behaviors observed in OECTs, they fall short in explaining several critical aspects of OECTs performance. These aspects include the asymmetric characteristics of the OECT’s on/off switching [45], the delayed response in the transient *I*_DS​_ [45], and occasionally slow switch-off speeds. The mobility of ions, which can move both perpendicular to the channel (as considered in the Bernards model) and along the channel under the influence of *V*_DS_​, significantly impacts the steady-state and transient behaviors of OECTs. Moreover, the Bernards model does not account for the potential barriers to ion transport within the electrolyte and the OMIEC channel. The model simplifies ion transport to be solely governed by RC dynamics (resistance and charging capacitance). However, in some OECTs systems, ion transport, especially in the crystal phase of the OMIEC, may need to overcome transport barriers, leading to decay and even non-volatile behavior.

#### Equilibrium Models

The capacitive model calculated the ion distribution and charge concentration based on the gate capacitance and the voltage applied at the gate. However, such models inherently assume that ion movement is restricted to a direction perpendicular to the channel’s length. This overlooks the possibility of mobile ions moving sideways within the channel, which is a direction parallel to the electric field generated by the drain potential, indicating a more complex ion transport behavior.

**Fig. 4:**
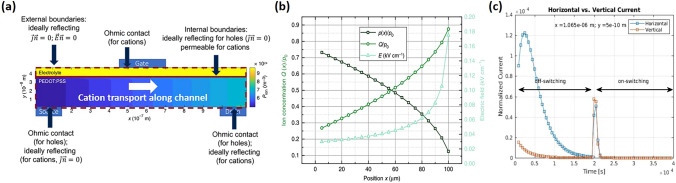
**a** Two-dimensional OECT drift–diffusion model, with green arrows representing cation flow directions within the channel. **b** The transverse ion concentration and hole concentration at equilibrium. (**a, b**) Reproduced with permission [30]. Copyright 2020, Kaphle et al. **c** Horizontal and vertical ionic currents in the transistor channel during off-switching and on-switching. Reproduced with permission [47]. Copyright 2022, Wiley–VCH

Neglecting lateral ion currents in capacitive models leads to a non-equilibrium ion distribution along the channel and hence a non-equilibrium hole and potential distribution along the channel as well. Kaphle et al. introduced a finite-element 2D drift–diffusion simulation by incorporating lateral ion currents along the channel [30] (Fig. 4a). This inclusion revealed that lateral ion currents lead to ion accumulation at the drain contact, significantly altering transistor behavior from predictions made by the Bernards model (Fig. 4b). Building on this improved model, Paudel revealed the importance of considering hole and ion concentrations along the transistor channel without averaging [46,47]. They show that lateral ion currents within the channel is the slow process that limit the switching speed [47] (Fig. 4c). Following a rapid drop in drain current due to ions being injected vertically into the transistor channel, ions redistribute inside the channel through lateral currents until the OECTs reaches a steady state. The rate of this redistribution, and consequently the relaxation of drain currents, can be slow, depending on factors like the applied drain potential, channel length, and the precise geometry of the OECTs. The inherent characteristic of mobile ions in OECTs, coupled with generally slow ion mobility, makes lateral ion transport a significant limiting factor for OECTs switching speed (Fig. 2) [32,36]. This is a key reason why, in many OECTs, the switching-on speed is much slower than the switch-off speed. It also explains why the time constant during switching-on is much larger than that during switching off, even though the ionic circuit remains unchanged.

Experimentally, Guo et al. explored the effect of lateral and vertical ion transport on the asymmetric switching speeds of OECTs [45]. Utilizing operando optical microscopy, they show that the device’s switch-on process unfolds in two distinct phases. Initially, a doping front moves from the source toward the drain. Subsequently, the channel, now partially doped, experiences a more uniform doping process. Conversely, the turn-off process is more straightforward, occurring in a single phase where dedoping kinetics show minimal variation across the channel, with the fastest dedoping observed near the source. This phenomenon likely contributes to the decay observed in the transient response of OECTs (Fig. 2), especially in systems with low ion mobility.

Other than lateral ion transports, the kinetics of doping and dedoping [48–50], and the impact of carrier density-dependent mobility could affect the transient switching speed [45]. Doping processes are inherently slower than dedoping, presumably due to the structural rearrangement of the polymer during doping [48]. Significantly, the mobility of carriers, which varies with carrier density (particularly in regions of high carrier density) [30,38,45], plays a crucial role in device dynamics. Friedlein et al. demonstrated that considering carrier density-dependent mobility provides a comprehensive understanding of steady-state and transient OECTs performance. The mobility-density relationship is given by:

4 $$\mu ={\mu }_{0}\cdot {\left(\frac{p}{{p}_{0}}\right)}^{\frac{{E}_{0}}{{k}_{B}T}-1}$$

where *μ*_0​_ is the mobility prefactor, *p*/*p*_0_​ represents the ratio of hole concentration to its zero-field value, *E*_0_​ the energetic width of the density of states tail, *k*_B_​ Boltzmann’s constant, and *T* temperature. This equation, reflecting the impact of energetic disorder in conjugated polymer materials, suggests enhanced turn-off speed in OECTs, where a significant decrease in both carrier density and mobility marks the initial stage of device turn-off.

In addition to the volatility that assumed in OECT models, hysteresis and even non-volatility is also often observed in OECT testing, and particularly in neuromorphic synapses and ECRAM. The role of ion transport energy barriers in the hysteresis and non-volatility of OECTs has been underscored by numerous studies, pointing to influences such as ion size, crystallization within the OMIEC, or intentionally introduced ion transport barriers [4,20,51–53], as well as the structural hysteresis induced by ion doping [39]. Despite these insights, few device models have comprehensively accounted for these factors in describing OECTs hysteresis phenomena. Bisquert et al. identified four distinct relaxation phenomena that contributing to the complex dynamics of hysteresis in OECTs: time constants related to electronic and ionic currents, vertical and lateral ion diffusion, and the effects of electrolyte resistance and film capacitance [54]. They also distinguish between capacitive and inductive hysteresis, associated with ion diffusion in the organic film, which manifest as counterclockwise and clockwise loops, respectively, in the transfer current [54]. Koch et al. reproduced the forward–backward hysteresis curves by developing a drift–diffusion simulation model that incorporates an incomplete ionization approach, leveraging Poisson–Boltzmann statistics for accurate simulation of charge densities, and electrostatic properties of OECTs. While these works clearly distinguished between the non-kinetic and kinetic regimes of hysteresis, the true origin of the non-kinetic hysteresis remains open for investigation [52]. Future theoretical studies on hysteresis and non-volatility behavior in OECTs need to consider additional electrical parameters into the equilibrium model. Specifically, considerations should include the doping and dedoping kinetics, ion transport energy barrier, especially given that structural changes under operational conditions could alter the energy barriers for ion transport.

### Dynamic and Morphological Changes During Transient and Characterization Methods

Models that describe the transient response and operation of OECTs typically assume static material and structural features. However, such models fail to consider the dynamic morphological and structural changes occurring within OMIECs during device operation. For example, BBL, which swells negligibly in electrolytes, undergoes a drastic and permanent change in morphology upon electrochemical doping, while keeping the molecular packing remains undisrupted, leading to exceptional mixed electron and ion transport despite lack of ion-coordinating side chains [55,56]. Similar structure and morphological changes have also been observed in other OECTs systems [36,57–59]. These changes significantly affect the dynamics of charge carriers and ions, making it essential to recognize and understand them for advancing OECTs design and materials. The dynamic nature of OMIECs implies that analyzing structure–property relationships at a single state or equilibrium is not sufficient. Research in this area should cover various conditions, adjusted through electrochemical potentials or electrolyte concentration changes. Establishing these relationships is a key objective for researchers, requiring a diverse set of characterization techniques. No single method can fully address all aspects of structure–property analysis, necessitating a multifaceted approach that includes device testing, scanning probe microscopy, scattering techniques, and spectroscopy to examine OMIEC structure, transport mechanisms, and ionic-electronic interactions across different scales.

**Fig. 5:**
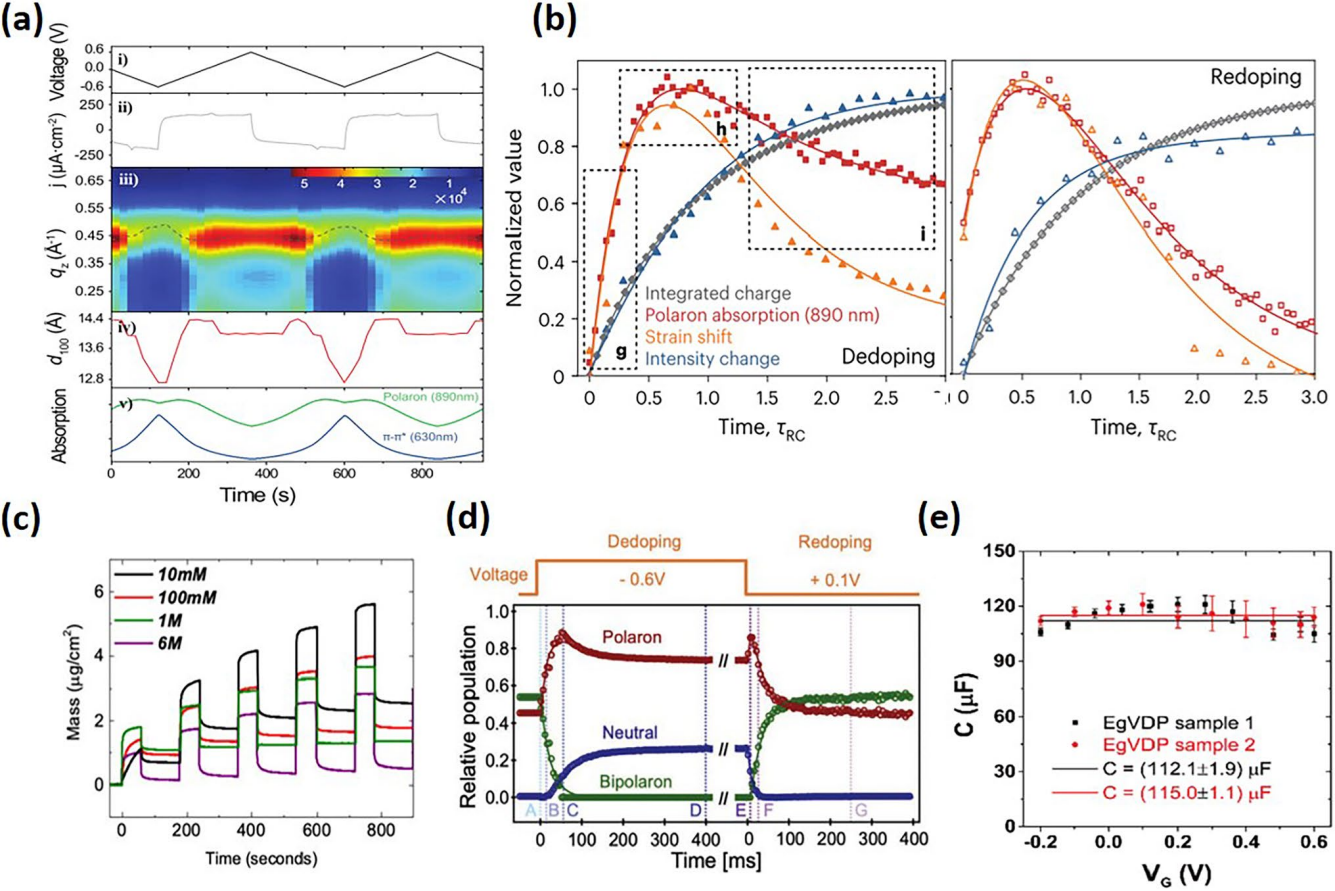
**a** Cycled linear potential sweeps during operando GIWAXS and UV–vis–NIR spectroscopy: (i) Potential profile, (ii) current density traces, (iii) out-of-plane scattering intensity color map, (iv) lamellar d-spacing, and v) 630 nm π–π* (*blue line*) and 890 nm polaronic (*green line*) absorption over time. The *dotted line* in (iii) serves to indicate the maximum lamellar scattering intensity at each time step. Reproduced with permission [48]. Copyright 2020, Wiley–VCH. **b** RC-normalized curves for the dedoping (*top*) and redoping (*bottom*) processes to show the order of polaron population kinetics (*red*), and the mesoscale-domain strain (*orange*), phase contrast (*navy blue*) and charge transport (*gray*) transients. Reproduced with permission [39]. Copyright 2024, Nature Portfolio. **c** The number of water molecules injected into the films at the end of the doping pulse at 0.5 V and remaining in the film upon the subsequent dedoping pulse applied at 0 V as a function of NaCl concentration. Reproduced with permission [60]. Copyright 2019, American Chemical Society. **d** Dynamics from the MCR deconvolution of the neutral, polaron, and bipolaron populations for dedoping and redoping. Reproduced with permission [49]. Copyright 2022, Wiley–VCH. **e** Changes of film capacitance upon different gate voltage. Reproduced with permission [61]. Copyright 2021, Wiley–VCH

Grazing incidence wide angle X-ray scattering (GIWAXS) has emerged as a crucial technique for real-time (in situ and in operando) studies of crystallization within OMIECs [48,62–69]. It provides insights into lattice spacings and allows researchers to link changes in electronic charge transport with crystalline alterations caused by environmental factors and doping. Observations from GIWAXS on the evolution of crystalline microstructure shed light on how doping influences ionic-electronic coupling, thereby enhancing conductivity in OMIECs. Such insights are vital for comprehending OECTs transient behaviors across different operational states. The complex interplay between ionic and electronic transport, especially with hydration and swelling effects in OMIECs, significantly influences device morphology and electronic properties. This interaction is further affected by the relationship between ions and conjugated polymer chains, leading to substantial modifications in the polymer’s local structure. Kukhta et al. investigated the structural dynamics of polythiophene derivatives upon doping with lithium bis(trifluoromethanesulfonyl)imide (LiTFSI) [70]. Doping was found to disturb the crystalline structure and penetrate both crystalline and amorphous domains, showing a preference for the amorphous areas, highlighting the importance of amorphous regions in supporting ionic transport. Cendra et al.’s work, integrating GIWAXS with other analytical methods like resonant soft X-ray scattering and ion transport studies, points to the formation of a percolated microstructure that benefits electronic transport but hampers ionic movement. They demonstrate how doping-induced interactions between crystallites, ions, and water cause notable lattice expansions and contractions, affecting electronic properties related to chain transport (Fig. 5a) [69]. Moreover, Flagg reported that doping P3HT with TFSI^−^ results in a pronounced crystal structure contraction and a stepwise increase in film mobility, associating these changes with charge carrier density alterations, suggesting that beyond a certain doping threshold, device mobility enhancements may be attributed more to amorphous regions than to crystal structure modifications [71].

In addition, GIWAXS studies have identified asymmetric rates of structural changes in materials, correlating directly with the transient behavior of polaron–bipolaron kinetics in polymers like PEDOT:PSS. The distinction between polaronic and bipolaronic charge carriers along the polymer backbone plays a crucial role in defining the transient structural behavior, suggesting that bipolaron population dynamics could limit the speed of devices. Further, X-ray photon correlation spectroscopy (XPCS) reveals fingerprint of the system microstate, allowing a direct and quantitative measure of a system’s evolution across different length scales and timescales. By using the grazing incident XPCS, Wu recently demonstrated unexpected coupling of charge carrier dynamics with the mesoscale order, where strain and structural hysteresis depend on the sample electrochemical cycling history under both adiabatic and non-adiabatic cycling conditions (Fig. 5b) [39]. Techniques including GIWAXS and moving front ion transport methods have shed light on the decreased ionic transport due to the formation of a percolated microstructure, which, while beneficial for electronic transport, impacts device performance [48,72]. GIWAXS has also been adopted in studying the texture of dry polymer films, enabling the examination of control over lamellar spacing and π–π stacking influenced by various side chains. This analysis helps correlate morphological alterations with OECTs performance, especially in understanding the effects of doping and water intake on polymer microstructure.

The electrolyte-swollen state of OMIECs is vital for comprehending structure–property relationships. Techniques such as Raman spectroscopy have been instrumental in investigating ionic-electronic coupling and the nature of electronic charging within conjugated polymers, highlighting the differences in electronic charges within ordered versus disordered domains. Integration of ex situ GIWAXS and in situ Raman spectroscopy has been applied to connecting hydration-induced microstructural changes with device performance, showing how anions infiltrate crystallites and the role of water in amorphous regions. Achieving an optimal balance of swelling in the OECTs channel can enhance ion penetration while minimizing structural deterioration, thereby maximizing transconductance and improving switching speeds [60,73].

Quartz crystal microbalance with dissipation monitoring (QCM-D) is another technique providing valuable insights into OMIEC films by tracking changes in oscillation frequencies and energy dissipation of a quartz crystal coated with an OMIEC film. Changes in frequency and dissipation relate to mass (or thickness) variations and the softening of the film, respectively. QCM-D studies have demonstrated the efficiency of ion-to-electron coupling in PEDOT:PSS films, revealing that electrochemical doping causes the film to absorb more ions than it expels, with water being drawn into the film alongside ions (Fig. 5d). Although this process can significantly swell the films without negatively affecting their performance, excessive water uptake during operation can be detrimental to electronic charge transport by irreversibly altering the film morphology. While increased hydration facilitates ion transport, it also reduces charge mobility, underlining the importance of optimizing film swelling through chemical design or adjusting device operating conditions [60,65,73,74].

Time-resolved spectroelectrochemistry can be applied for analyzing the behavior of polaron and bipolaron formation dynamics in OECTs materials. This method utilizes both steady-state and time-resolved approaches to differentiate between the neutral, polaron, and bipolaron states within these materials. Rebetez’s studied the doping and dedoping kinetics in PEDOT:PSS OECT, highlights that the doping level is governed by thermodynamic equilibria, which are influenced by Gibbs free energy (Fig. 5e). The dedoping and redoping processes are understood through kinetic modeling as sequential first-order electrochemical reactions, emphasizing the role of enthalpy and entropy. Notably, it has been found that ion diffusion rates surpass those of these redox reactions, indicating that ion diffusion might not limit device dynamics under certain conditions [49].

Electrochemical impedance spectroscopy (EIS) is another essential tool, stands out for its ability to separate and define ionic and electronic transport in OECTs through the measurement of frequency-dependent impedance from current–voltage small signal analysis. This enables the determination of ionic and electronic mobility, conductivity, and the degree of ionic-electronic coupling. The complex impedance spectra of OMIECs require transmission line models for thorough analysis. EIS is effective in quantifying the ionic-electronic coupling, showcasing it as either frequency or voltage-dependent capacitance, thus shedding light on the complex relationship between ionic and electronic components within the devices (Fig. 5f) [61].

## Transient Response Optimization Strategies of Volatile OECT

OECTs typically operate within the frequency range of 10–100 kHz, in contrast to FETs which can function up to the MHz range. The response time of OECTs is constrained by the velocity of ionic or electronic charge carriers. To reduce response time, strategies have been implemented based on the Bernards model. These strategies include increasing the mobility of charge carriers and ions, reducing the overall capacitance of the channel and electrodes, and minimizing the distance for ion transport, all contributing to quicker electrical switching speeds. Although it is possible to achieve faster device responses than the ionic charging circuit speed by employing specific measurement methods, such as operating the OECTs only under conditions described by ${V}_{\text{STEP}}=-\frac{{L}^{2}}{2{\mu }_{\text{OECTs}}{R}_{\text{S}}{C}_{\text{Ch}}}$ [43], such method maybe however not be practically applicable. Therefore, here we will focus on the strategies that improve the inherent switching speeds.

### Materials Effects

In general, the choice of materials and their structure in OECTs—including microstructure, packing mode, and order significantly affects charge mobility and capacitance C^*^, as well as ion mobility [70]. While dense and highly crystalline phase offer high mobility, enhancing electron conduction, this may hinder the swelling necessary for effective ion injection and achieving high capacitance. On the other hand, the amorphous phases facilitate faster ionic transport, which is essential for fast OECTs operation. However, achieving high charge carrier mobility with efficient ion penetration and transport often presents a conflict; increased molecular packing and crystallinity enhance electrical conductivity but may obstruct ion movement.

To overcome these challenges, it is important to develop conjugated polymers and balanced morphology that benefits both ion and electron transport. Hydrophobic semiconducting polymers with high mobility can block ion penetration, necessitating strategies to integrate hydrophilic side chains into the polymer. Both modifications of the polymer backbone and its side chains are crucial for achieving high-performance in these devices [9,35,75–77]. In this review, we will provide a brief introduction of side-chain modifications, as they are more relevant to ionic transport. For more on backbone modifications and detailed information on side-chain engineering, especially from the perspective of materials chemistry, readers are encouraged to refer to more specific papers.

**Fig. 6:**
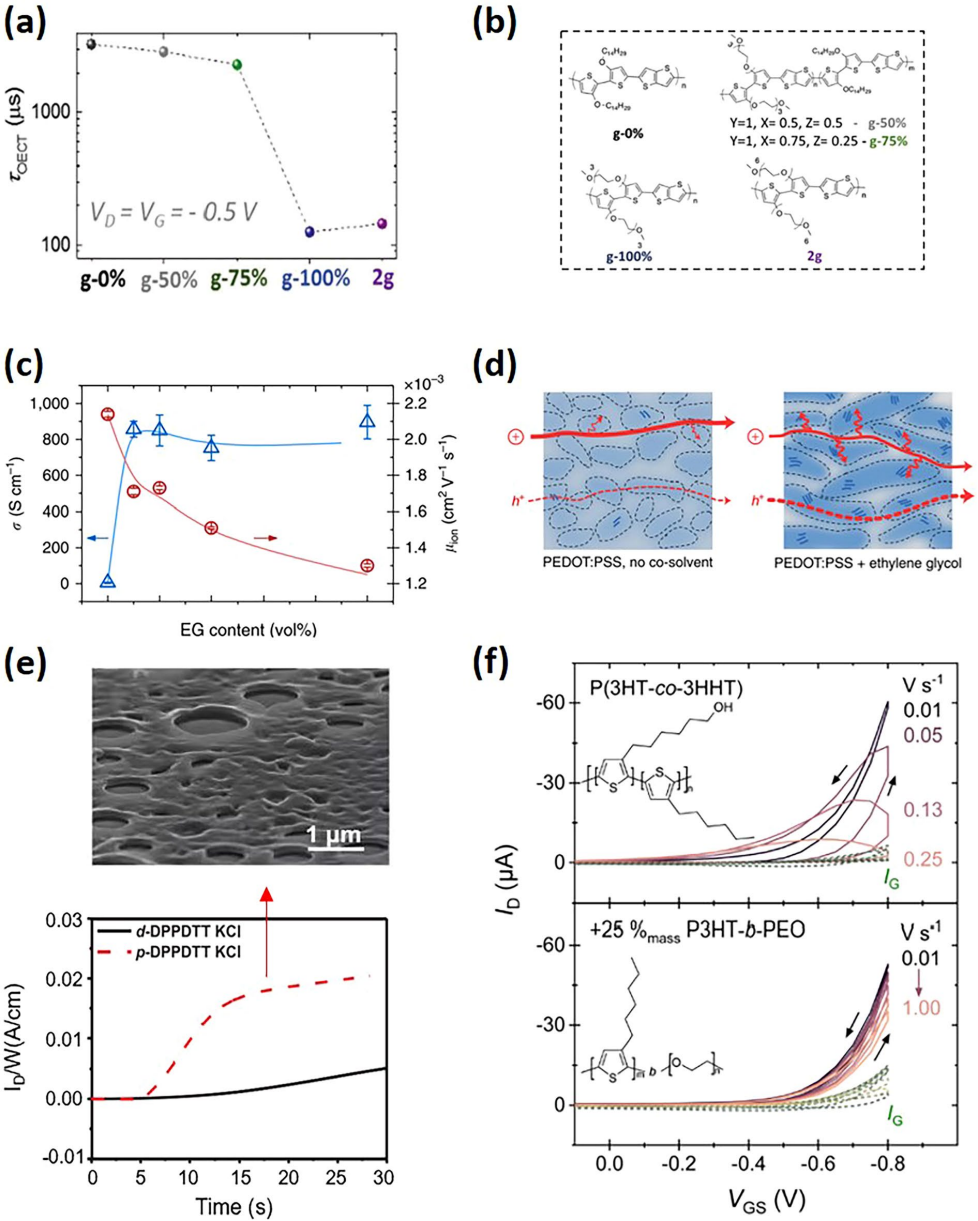
**a** *τ*_OECT_ of OECTs’ channel materials in the polymer series with increasing contents of EG side chains, with the channel materials listed in **b**: g-0% is p(a2T-TT), g-50% and g-75% are random copolymers with p(g2T-TT):p(a2T-TT) ratios of 50:50 and 75:25, respectively, and g-100% is p(g2T-TT); 2 g is the homopolymer with longest EG side chains. (**a, b**) Reproduced with permission [73]. Copyright 2020, Wiley–VCH. **c** Electronic conductivity (blue), K^+^ ion mobility (red) as a function of EG content. **d** A schematic illustration of the morphological changes and associated transport of ions and holes. (**c, d**) Reproduced with permission [88]. Copyright 2016, Rivnay et al. **e** OECT switching properties of the DPPDTT polymer films (dense, porous). The red arrow points to the porous film SEM images for p-DPPDTT. Reproduced with permission [91]. Copyright 2021, Wiley–VCH. **f** Transfer curves of P(3HT-co-3HHT) and its blend with P3HT-b-PEO. Blending with P3HT-b-PEO leads to less hysteresis in current traces. Reproduced with permission [92]. Copyright 2023, Royal Society of Chemistry

Recent studies have highlighted a tradeoff between enhancing the polymer’s hydration to facilitate ion conduction and preserving efficient charge transport, both closely related to the polymer’s morphology. For instance, a glycolated version of P3HT, poly(3-thiophene-2,5-diyl) (P3MEEMT), showed quicker anion injection kinetics than P3HT, largely unaffected by the anion type [37,71]. P3MEEMT’s crystal lattice could expand in solution, allowing ions to move freely within the material. The level of crystallinity significantly impacted hydration effects on the connectivity of crystalline domains within the film, thus affecting OECTs charge carrier mobility. Higher crystallinity in P3MEEMT led to reduced hole mobility as hydration disrupted connections between crystalline domains. Another case involved systematically altering p(g2T-TT) and its analogs to increase hydrophilicity [73,78]. By adjusting the glycolation level of the side chains from fully alkylated to fully glycolated copolymers, notable improvements in transconductance were achieved, thanks to enhanced ion movement encouraged by polymer swelling (Fig. 6a, b). However, an excessive increase in hydrophilicity, as observed in the polymer “2g” with extended hexakis glycol side chains, resulted in performance decline. This drop was linked to the excessive hydration causing crystalline regions to separate, diminishing the polymer’s charge capacity and hole mobility. Interestingly, the “2g” film, despite swelling more, showed lower transconductance, mobility, and switching speed compared to the optimally glycolated p(g2T-TT), demonstrating the critical balance between hydrophilicity and electronic properties for the best OECTs performance.

The electrolyte also significantly influences ionic kinetics. Commonly used electrolytes include water-based, room temperature ionic liquids (RTILs), or comprise more complex mixtures. These mixtures can result in a variety of consistencies, ranging from liquid solutions to gels, including hydrogels and crosslinked solid electrolytes [79,80]. Among these, aqueous electrolytes typically yield a high transconductance and faster speed, despite at the cost of a lower on/off ratio. Both the concentration and the composition of the electrolytes are crucial, as evidenced by the observed direct dependency of the impedance of OECTs on the concentration and the nature of various electrolytic cations (Li^+^, Na^+^, Cs^+^, Rb^+^, K^+^, Ca^2+^). For example, it has been reported that the device’s sensitivity varies with the size/mass of the cations at low frequencies and with the valency and ionic conductivity at high frequencies [81].

In addition to channel materials and electrolytes, the choice of electrode materials is paramount in affecting the performance of OECTs, including their transient response. Electrodes can be divided into polarizable and non-polarizable categories [82]. In perfectly polarizable electrodes, no actual charge transfer occurs at the electrode/electrolyte interface, with the current across the interface being a displacement current, rendering the electrode’s behavior capacitive. Platinum and gold are considered nearly perfect polarizable electrodes, exhibiting significant charge separation at the electrode–electrolyte boundary, which electrically parallels the interface to a capacitor. This capacitive effect could introduce additional capacitance, potentially hindering the ion injection process. In practical scenarios, gate electrodes are often modified with nanoparticles (e.g., Pt nanoparticles) or other nanomaterials (e.g., graphene, reduced graphene oxide) to enhance their surface-to-volume ratio and sensitivity toward specific analytes [83]. Additionally, crosslinked enzymes are frequently employed to catalyze targeted reactions at the gate, and biocompatible polymers (such as chitosan or Nafion) are used to immobilize the enzymes (the embedded holes within the polymer films allow unimpeded transport of analytes), enhance the detection limit and sensitivity of OECTs-based biochemical sensors [84,85]. Therefore, a balance between sensitivity and reduced response speed is essential.

Conversely, source/drain electrode materials have been recognized for their significant impact on the functionality of organic electronic devices, with their effects on OECTs only recently gaining research attention. Gold remains the predominant material for source/drain electrodes in OECTs, closely matching the highest occupied molecular orbital (HOMO) level of most *p*-type organic materials, like PEDOT:PSS. However, a contact resistance persists, affecting device performance [86,87]. Ersman et al. demonstrated improved device characteristics with the introduction of a carbon conductor layer atop PEDOT:PSS at the drain electrode, which facilitates faster switching times from off to on by mitigating the effects of a reduction front extending into the PEDOT:PSS contact [44]. Comprehensive microscopic models are necessary for a deeper understanding of charge injection dynamics in the presence of high ion concentrations at the source and drain electrodes, guiding the selection of materials for optimized contact.

### Morphological Effects

Through comprehensive investigations into the effects of doping, annealing, and the application of solvents and ionic liquids, researchers have been able to fine-tune the balance between ionic and electronic transport. While structures that are highly ordered and flat are conducive to charge transport, rougher, less ordered, and porous structures facilitate ion penetration [67,88–90]. Modifying the morphology presents a promising avenue for boosting OECTs performance, albeit it has been explored to a lesser extent than material synthesis.

Various methods have proven successful in optimizing OMIEC morphology, same as the morphology control methods adapted for other organic electronic devices. Studies by Flagg et al. on the hydration dynamics of annealed versus unannealed films revealed distinct effects on mobility and swelling following electrochemical doping [37]. Films that were annealed and more crystalline showed higher mobility before interacting with electrolytes but experienced a reduction in mobility upon doping due to increased film heterogeneity. However, post-annealing treatments, which typically enhance mobility in OFETs within crystalline areas, can impair performance in these polymers by reducing film order.

Additives have been extensively utilized in organic solar cells to tailor morphology and control crystallinity, thus enhancing molecular ordering and electronic properties [93–95]. Similarly, the addition of additives to conjugated OMIECs can aid in improving both electronic and ionic transport, thereby enhancing OECTs performance. Taking PEDOT:PSS as an instance, co-solvent additives are frequently incorporated to boost the film’s performance, including polar solvents like ethylene glycol (EG), dimethyl sulfoxide (DMSO) [94] to increase conductivity, and dodecyl benzene sulfonic acid (DBSA) [96] to modify film-forming properties. This additive strategy is often combined with post-treatment methods such as post-annealing or post-solvent annealing. For example, an increase in EG content has been shown to result in slightly closer π-π stacking among different polymer chains and a rise in crystallite size. This growth in domain sizes led to an increase in the heterogeneity of the PEDOT:PSS cores and PSS-rich matrices that constitute the films’ microstructure. Although coarser morphologies were detrimental to ionic charge carrier mobilities (μ_ion_ ~ 2.2 × 10^−3^ cm^2^ V^−1^ s^−1^ for blends with 0 v/v% EG to ~ 1.3 × 10^−3^ cm^2^ V^−1^ s^−1^ for those with 50 v/v% EG), they significantly enhanced electrical conductivity from 6 to 800 S cm^−1^ (Fig. 6c, d) [97]. Additionally, the use of a “bad solvent” like acetone has been reported to modulate the microstructure and morphology of P-90 films in a manner that supports both ionic charging and electronic charge transport [98].

Doping polymer films with ionic liquids represents a potent method. For instance, within the conjugated polyelectrolytes of PEDOT:PSS, the ionic liquid [EMIM][TCM] is incorporated as a third component. This addition diminishes the interaction between PEDOT chains and PSS groups, facilitating a closer stacking of PEDOT to form a fibrillar morphology. Consequently, PEDOT chains coalesce into a 3D fibrillar network, enhancing ion penetration and achieving a remarkably high transconductance of approximately 7100 S m^−1^ alongside a rapid transient response of 3.9 ms [99]. However, the strong affinity of the ionic liquid introduces a high polarity, sustaining a large current over prolonged duration.

In addition to co-solvent additives, the interaction of salts with the polymer backbone can also enhance electronic charge carrier mobility, ion uptake, and influence morphology and molecular packing [100]. Schmidt et al. discovered that the smaller tetramethylammonium (TMA^+^) counterion leads to increased aggregation and π-stacking of polythiophene semiconductor (PTHS) chains compared to larger counterions such as tetrabutylammonium (TBA^+^) and tetraethylammonium (TEA^+^) [101]. This aggregation facilitates the formation of PTHS:TMA films exhibiting improved oxidation efficiency and reversibility. Thus, OECTs utilizing PTHS:TMA as the active layer outperform those with PTHS:TBA and PTHS:TEA, showcasing higher transconductance and faster switching times. In a related investigation, Paterson demonstrated that introducing a Lewis basic n-dopant, tetra-n-butylammonium fluoride (TBAF), to the semiconductor P-90 improves electron mobility, ion uptake, and storage, alongside generating a microstructure that supports more straightforward ion penetration and migration [102]. This addition also leads to smoother film morphology with decreased surface roughness. Moreover, the authors hypothesize that TBAF might eliminate the grain boundary, densify the semiconducting layer, and enhance ionic transport within the polymer film.

To optimize ion transport and charge transport in OECTs, researchers have ventured novel morphology beyond the simple amorphous-crystalline phase balance in dense film [103–106]. Huang, for instance, adopted the breath figure method, a technique inspired by natural processes, to fabricate porous organic semiconductor-insulator blend films [91,107]. These films feature textured layers and uniform nanopores that enhance surface roughness and depth throughout the film thickness, facilitating the ion/polymer interactions, and thereby significantly reducing response times compared to dense films (Fig. 6e). However, one should note that despite these improvements, the transient speeds of porous OECTs still fall short of the levels seen in state-of-the-art fast devices [91,108]. The present porous morphology designs mainly enhance vertical ion injection, with minimal impact on lateral ion transport along the channel. Future research is encouraged to develop new strategies for porous morphology that could improve both vertical and lateral ion transport.

Blending with other materials that strongly swells when in contact with the electrolyte is also an effective approach to augment the transient performance of polymeric mixed conductors without substantial chemical modifications. For instance, blending PEDOT:PSS with PEO improves both electronic and ionic conductivity, as the interaction between polyethylene oxide (PEO) and PSS leads to closer PEDOT stacking [109]. The enhancement in ionic conductivity may stem from the efficient swelling of PEO materials, facilitating a greater ion accommodation capacity. Another example is that Barker demonstrated that a 75:25 blend of P3HT: P3HT-b-PEO operates more efficiently in aqueous electrolytes than neat P3HT, which exhibits no transistor behavior (Fig. 6f). This enhancement is attributed to the block copolymer’s ability to restrict large-scale phase separation and induce partial vitrification of the active layer, ensuring a higher doping-eligible polymer fraction. This is in contrast to systems blending P(3HT-co-3HHT) with a PEO homopolymer, which tend to fully phase separate, allowing both components to crystallize freely [92].

Similarly, another strategy to address ion migration challenges in hydrophobic conjugated polymers involves blending with other ion transport materials. Metal–organic frameworks (MOFs), with their ordered pore size, high pore volume, and large specific surface area, offer a unique combination of inorganic and organic material advantages [110,111]. For instance, ion-conductive vertical nanopores formed within the 2D c-MOFs films lead to more convenient ion transfer in the bulk than the dense film [110]. Also, Hsu et al. show that by incorporating MOF-525 into the channel material reduces the turn-on time of the PBTTT-C14 device from 28.75 to 2.56 s and the turn-off time from 2.02 to 1.33 s, with the more considerable improvement in turn-on time attributed to the slow ion transport nature of PBTTT-C14 [111]. Key areas for further investigation include the MOF pore sizes relative to ion sizes, film morphology alterations, roughness, and ion transport within MOFs, critical for elucidating the mechanisms behind these performance enhancements.

### Geometry Effects

The geometry of the channel, encompassing its volume and the overall device structure, as well as the morphology of the conducting film, significantly impacts the transient response speed of OECTs. Reducing the film thickness or the dimensions (width or length) of the device can effectively decrease the transit time constant ($\tau$~*RC*), since the capacitance is directly proportional to the channel’s total volume [27]. This adjustment reveals a balance between transconductance (*g*_m_) and response time, indicating that the device’s geometry must be finely tuned to meet the requirements of its intended application. For example, designs aimed at high signal amplification may prefer larger *L/W* ratios, sacrificing bandwidth, whereas applications demanding rapid response times might select smaller *L/W* ratios [4,29]. The device’s overall capacitance is actually composed of two series capacitors (${C}_{eq}\equiv \frac{1}{1/{C}_{G}+1/{C}_{CH}}$): one at the gate/electrolyte interface (${C}_{G}$) and another at the electrolyte/channel interface (${C}_{CH}$) [82]. This arrangement affects both the transconductance and the response speed of the OECTs, particularly in cases where applications utilize a small polarizable gate electrode, its effect on the equivalent capacitance becomes significant.

#### Planar Structure

**Fig. 7:**
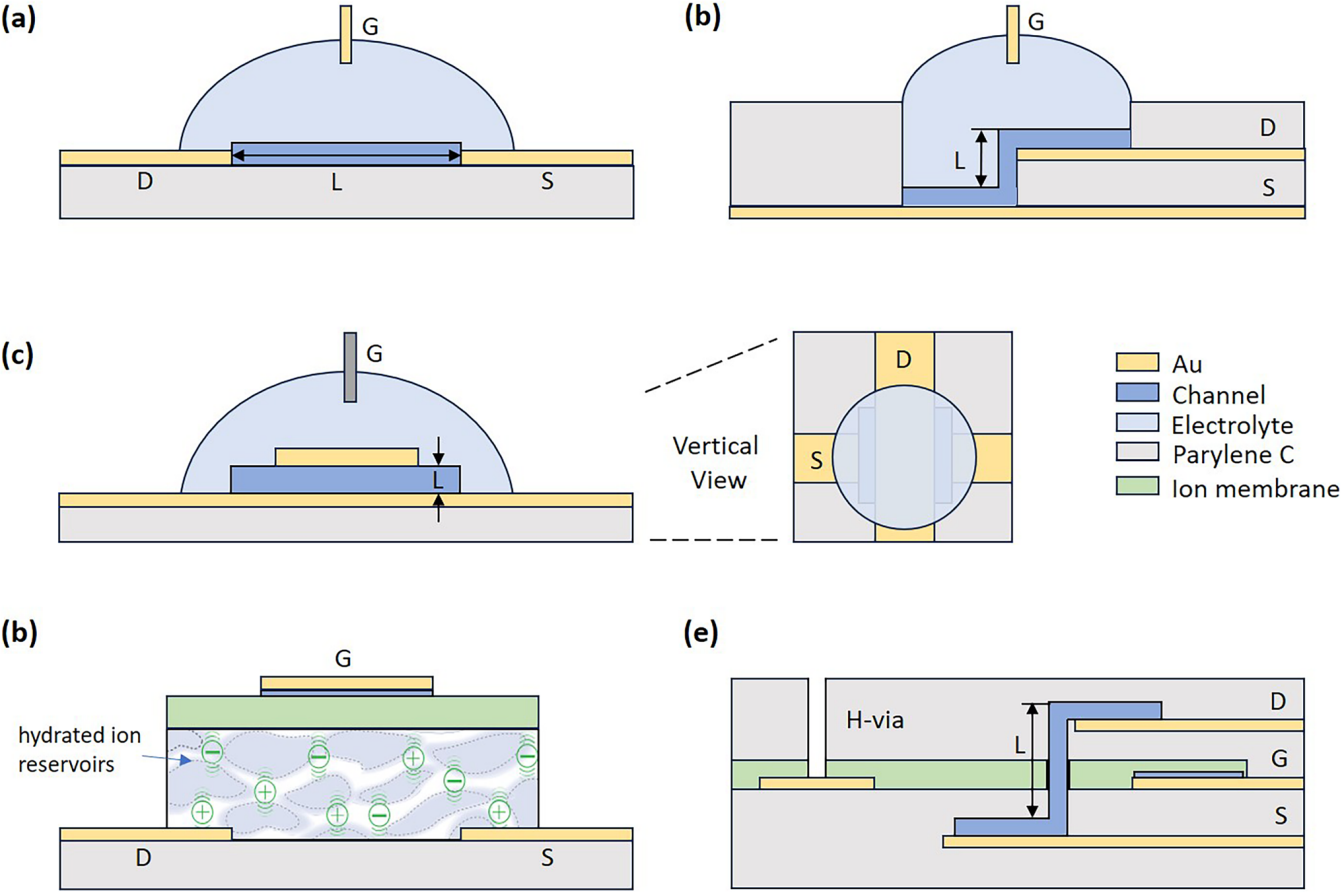
Diagram of OECT device structures. **a** Planar structure. **b, c** Vertical structure. **d** IGT. **e** Vertical IGT

The planar structure is the most common form of OECTs, often exceeding 5 μm in length due to fabrication constraints, and represents the standard model for these devices (Fig. 7a). Despite fast doping and dedoping kinetics at the channel level, planar OECTs face limitations in transient response speed due to lateral ion transport along the channel, especially during the switch-on phase [47]. In contrast to the slow switch-on speed, the switch-off phase is typically much faster, frequently up to ten times quicker than the switch-on speeds.

#### Vertical Structure

Vertical OECTs leverage the intrinsic bulk doping characteristic of OECTs, unlike the interface doping seen in FETs, allowing for a simpler fabrication process of vertical structures [29]. The change from planar to vertical structure approach effectively reduces the channel length *L*, addressing the challenges posed by slow ion migration and bulky device geometry that contribute to low transient speeds. Transitioning from planar to vertical structures not only has the potential to decrease the overall size of the devices, but also to improve their transconductance, response times, and stability [112–114].

Donahue et al. introduced the first vertical OECTs structure, featuring vertically stacked contacts that enable channel length *L* resolutions down to 450 nm, significantly smaller than what is typically achievable through photolithography [115], allowing for smaller device footprints while potentially enhancing their electronic properties (Fig. 7b). Furthermore, Kleemann et al. proposed a novel 3D vertical organic transistor structure that operates volumetrically, in contrast to the traditional 2D interface of FETs [116], which improve the amplification or speed properties of OECTs by modifying the channel material and geometry.

However, the reduction in *L* is constrained by the thickness of the electrodes and insulating layers. To address this limitations, Huang and Wang et al. proposed simple sandwich designs, where the channel is enclosed between bottom and top electrodes, reducing *L* to below 100 nm (Fig. 7c). This significantly increases the width-to-length (*W/L*) ratio, leading to notable improvements in response times and on/off current ratios [22]. Comparative analysis between vertical and planar OECTs indicates that vertical structures can achieve quicker switching times and higher on/off ratios due to their reduced channel dimensions and more efficient ion transport. For example, volatile switching times for vertical OECTs (vOECTs) are significantly shorter than those of planar OECTs with comparable footprints [4]. Additionally, reducing the thickness of the OMIEC film to 70 nm has been shown to allow cutoff frequencies between 1.3 and 1.7 kHz for PEDOT:PSS layers. Nonetheless, these figures fall below theoretical predictions, highlighting the complexities involved in optimizing vOECTs performance due to additional parasitic resistance and capacitance. Based on vertical structure, Moon et al. achieved operation frequencies of at least 12 MHz in a PEDOT:PSSH vOECTs by using EMIMTFSI ion gel as the electrolyte and through optimized device geometries, marking a significant advancement [112].

#### Internal Gated Transistors (IGT) Structure

Vertical structuring presents a significant advancement in achieving higher operational speeds and greater amplification for OECTs by optimizing the device geometry. However, it faces challenges such as the speed of ion migration and the distance ions must travel to the doping sites. Spyropoulos et al. introduced a novel design known as the IGT structure. This design incorporates mobile ions within the conducting polymer channel, enabling both volumetric capacitance and reduced ionic transit time (Fig. 7d). The IGT’s channel comprises PEDOT:PSS combined with d-sorbitol. PEDOT:PSS, known for its biocompatibility, stability, and high conductivity, facilitates efficient ion-to-electron conversion. d-sorbitol, a hydrophilic sugar alcohol, aids in water molecule uptake, creating an “ion reservoir” within the conducting polymer and enhancing ion mobility. The addition of d-sorbitol also boosts the conductivity of PEDOT:PSS by extending PEDOT-rich domains, a benefit similarly observed with solvent additions like ethylene glycol [117,118]. This IGT design achieved *τ*_on_ and *τ*_off_ of 2.6 ms with a *L* of 12 μm, *W* of 5 μm, and a PEDOT:PSS thickness of 200 nm. Notably, the transient responses of such IGT designs are in the range of hole mobility (0.1–10 cm^2^ V^−1^ s^−1^) rather than ion mobility, leveraging the higher hole mobility of conducting polymers for increased operation speed. A specific high-temperature annealing process was utilized to enhance crystallization and form microstructures conducive to faster hole transport and higher conductivity, achieving a cutoff frequency of over 160 kHz.

Expanding upon this, Cea et al. developed the vertical internal ion-gated organic electrochemical transistor (vIGT) [24], which features a vertical channel and a miniaturized hydration access conduit (Fig. 7e). This architecture enables operation in the megahertz signal range within densely packed integrated arrays without crosstalk. The vIGT achieved a 900 ns response time in a channel with dimensions *W/L* = 5.0/0.8 μm and a thickness of 100 nm, with the channel length being 100 nm. This represents a significant advancement toward the integration of OECTs into digital electronics and complex logic circuits.

**Fig. 8:**
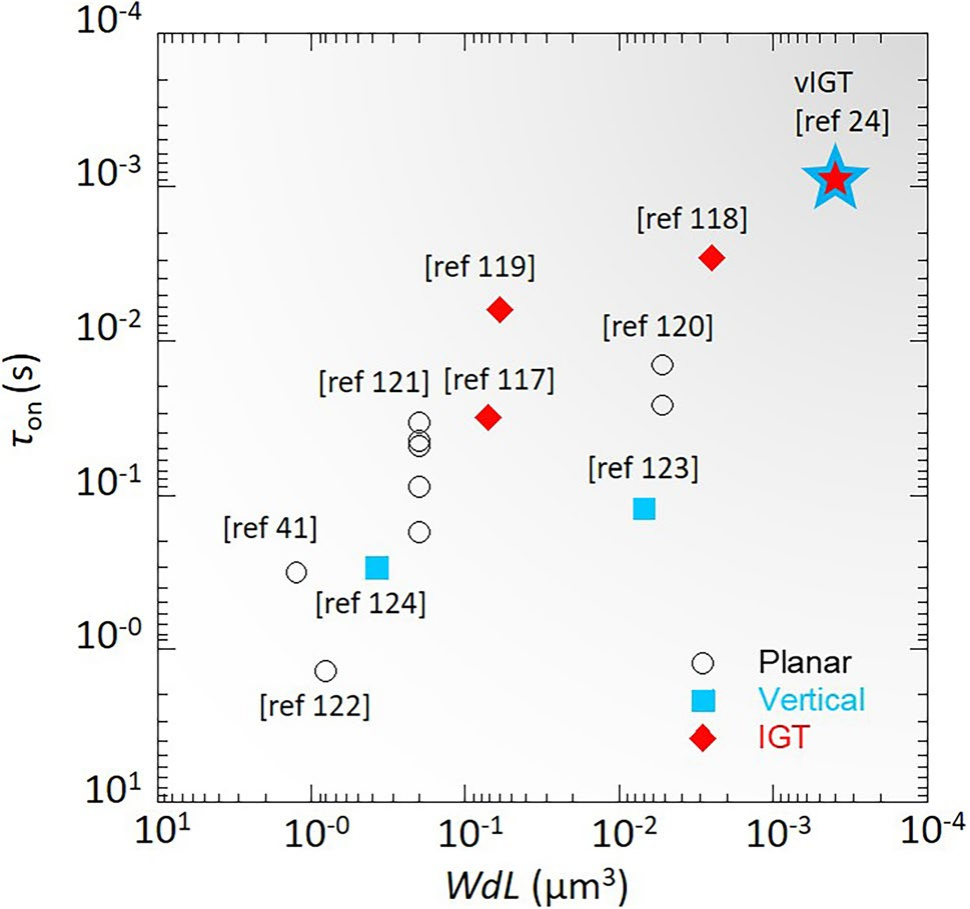
Transient response speed of OECT devices with different structure of PEDOT:PSS and its derivatives. All data were extracted from references [24,41,117–124]

To comprehensively compare the impact of different OECT device structures on transient switching speed, we collated data from the literature concerning PEDOT:PSS related to planar, vertical, IGT, and vIGT structures, focusing on response speed in relation to channel volume (Fig. 8). The overall trend shows the time constant τ decreases with a reduction in channel volume, almost linearly. Among these device structures, IGT, especially vIGT, exhibits the fastest response speed, primarily due to their sufficiently small channel volumes. On the other hand, it appears that as the channel volume decreases and the device size approaches sub-μm, the reduction in τ is not as significant as expected. This may be attributed to the more pronounced effects of parasitic capacitance and electrode area in micro fabricated devices. It is important to note that our choice to focus on PEDOT:PSS is because, currently, it is the only material applied across these various device structures. Considering the high ionic mobility of PEDOT:PSS [32,33], the impact of different device structures on channel length, and consequently on ionic lateral migration that affecting transient response speed, may not be fully evident. Systematic studies across a range of different OMIEC material types and device structures are needed in the future.

## Non-Volatility in OECTs

In the human brain, the neural network comprises approximately 10^11^ neurons, interconnected by 10^15^ biological synapses. These synapses facilitate neurotransmitter exchange across pre- and postsynaptic membranes, essential for information processing. Synaptic behavior exhibits two forms of plasticity: LTP and STP, categorized by the duration of synaptic modifications. LTP can persist for hours to years, whereas STP diminishes within minutes [125]. Further subdivision includes short-term facilitation (STF), short-term depression (STD), long-term facilitation (LTF), and long-term depression (LTD), for STP and LTP, respectively. STP plays a vital role in spatiotemporal information processing in biological systems, while LTP is associated with learning and memory [21].

**Fig. 9:**
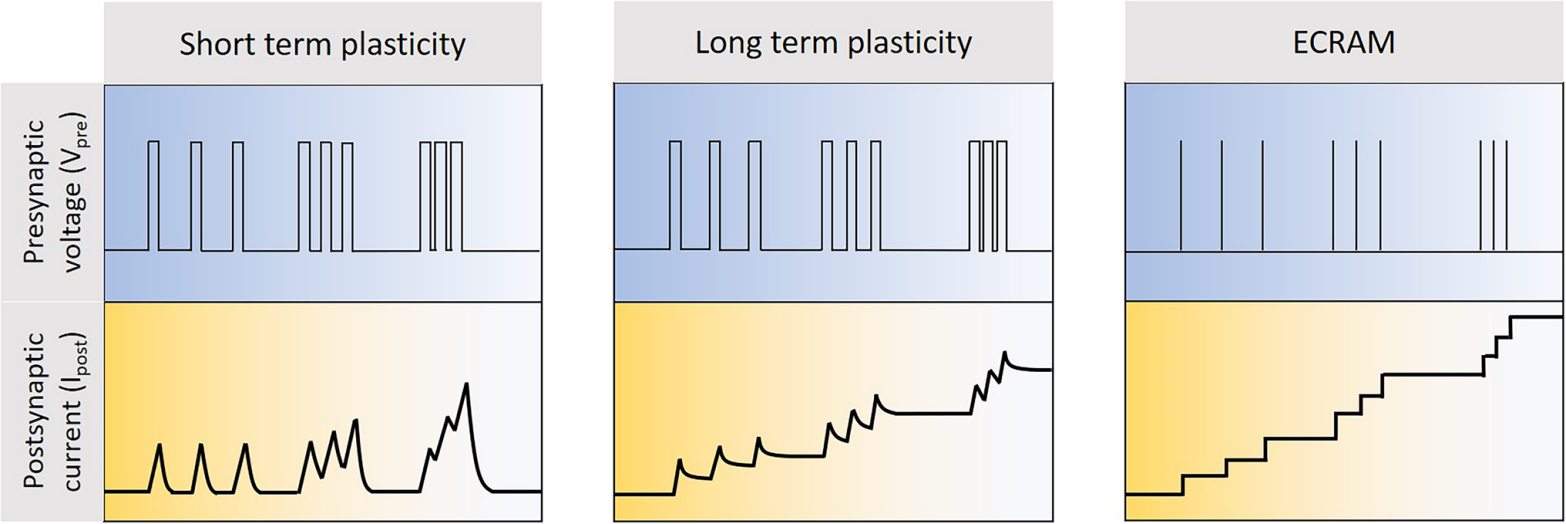
Schematic illustration of presynaptic voltage and corresponding postsynaptic current of STP, LTP and ECRAM under gate voltage pulses of different frequencies

Ion-gated transistors have demonstrated foundational electrical transport behaviors of artificial synapses, including STDP, spike-rate-dependent plasticity, and both short- and long-term potentiation by transmitting electronic signals through ionic charge migration under a gating field, analogous to synaptic operations in the brain [2,4,21,34] (Fig. 9). ECRAM devices share a similar architectural framework with OECTs, notably utilizing a high-capacitance, polarizable gate electrode to achieve device non-volatility. Contrary to the fast-switching speeds focused on in the previous section, the ion dynamics in these devices, especially when switched off, should be slow to emulate synaptic functions effectively. This slow ion motion allows transistor channels to exhibit continuous and variable conductance based on the voltage history, facilitating state retention. In this section, we review and analyze the foundational physical mechanisms and empirical evidence for slow ion dynamics. Additionally, we examine methods for controlling ion dynamics from the perspectives of materials, morphology, and device structure.

Numerous ion-permeable polymers have been employed in state-of-the-art neuromorphic devices, including conducting polymers like PEDOT:PSS [19,51,126,127] and semiconducting polymers such as thiophene-based semicrystalline polymers P3HT [128,129], p(g2T-TT) [20], PBDTTT-C-T [130], and donor–acceptor polymers FT4-DPP [131]. These materials contribute to volumetric capacitance and additional charge carrier induction when ions penetrate the channel. From the materials point of view, the trapping of ions within this layer, whether short- or long-term, could be attributed to strong crystallinity induced structural hinderance for ion transport, structural changes upon ionic doping, and ionic doping-induced electrochemical reactions [128]. For instance, Gkoupidenis and colleagues introduced a PTHF-based PEDOT derivative, PEDOT:PTHF. This material underwent a structural transformation upon the application of a high reduction potential, necessitating an oxidation potential of opposite polarity to revert this change. Consequently, PEDOT:PTHF-based OECTs demonstrated non-volatile behavior, capable of operating within both short-term memory and long-term memory regimes. Notably, the devices exhibited a lasting increase in synaptic efficacy following repeated stimulation, indicative of long-term synaptic phenomena. Other advancements in materials aspects were reported by Gerasimov et al., who developed an adaptive synaptic OECTs through the electropolymerization of a self-doped conjugated monomer, sodium 4-(2-(2,5-bis(2,3-dihydrothieno[3,4-b][1,4]dioxin-5-yl)ethoxy)butane-1-sulfonate (ETE-S), serving as the channel [132]. Long-term potentiation and depression properties were achieved by electropolymerization and electrochemical overoxidation of the channel material, under gate voltage spikes of 0.5 and 2 V for durations of 1 s, respectively.

**Fig. 10:**
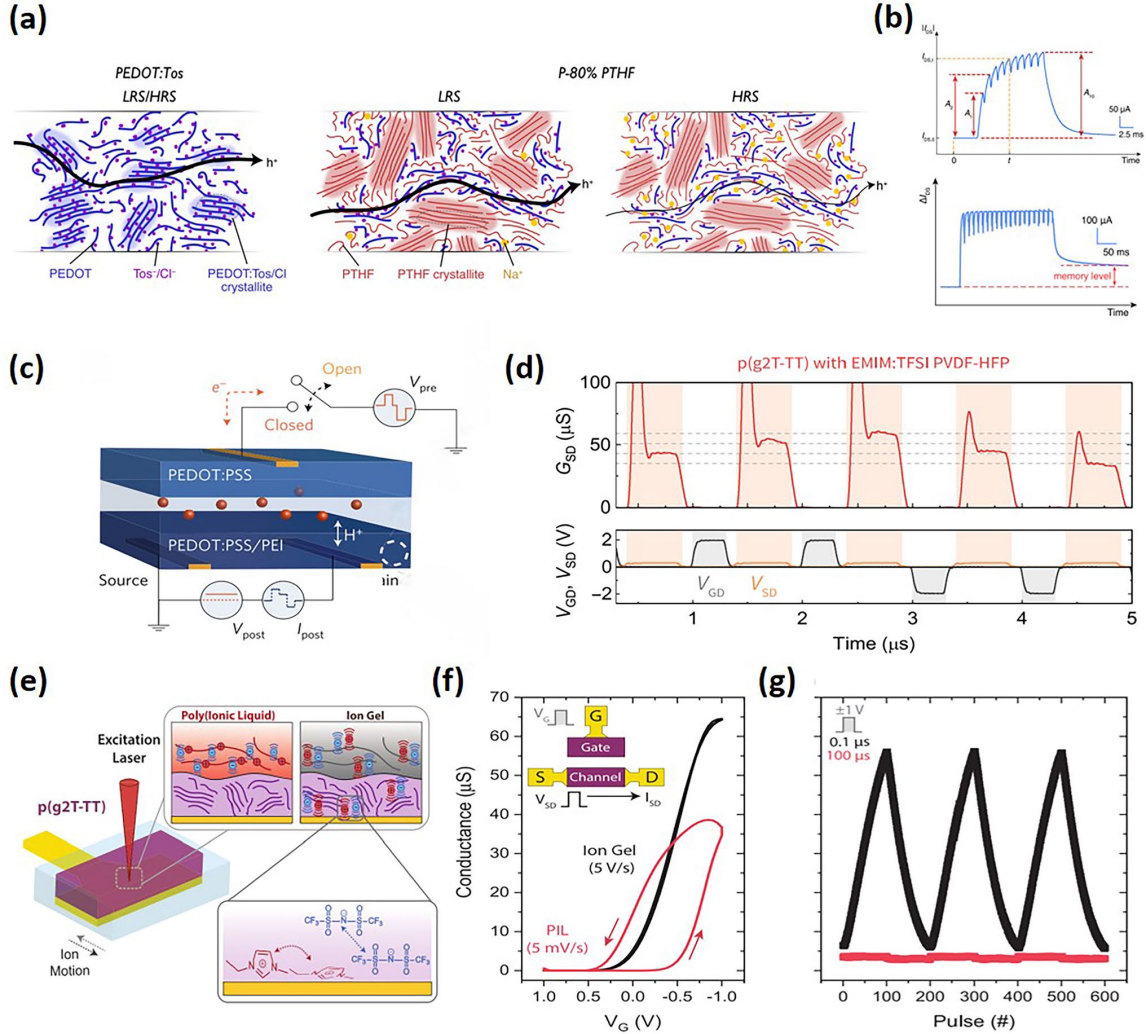
**a** Cartoon of the microstructure and composition of PEDOT:Tos and P-80% PTHF in low resistance state and high resistance state illustrating the mechanism of non-volatility. **b** Short-term malleability, as well as the transition from short-term to long-term malleability after a series of continuous gate voltage pulses. (**a, b**) Reproduced with permission [34]. Copyright 2021, Ji et al. **c** Sketch of the electrochemical neuromorphic organic device structure using Nafion as electrolyte. Pre- and postsynaptic layers are separated by an electrolyte layer transporting ions/protons (red spheres). Reproduced with permission [51]. Copyright 2017, Nature Portfolio. **d** Transient response of p(g2T-TT) EMIM:TFSI PVDF-HFP device potentiation and depression, spike-and-recovery feature at each read process. Reproduced with permission [20]. Copyright 2020, American Association for the Advancement of Science. **e** Schematic of ion distribution in p(g2T-TT) device measured with PIL and ion gel. **f**–**g** sweeping and pulsing characteristics (+ / − 1 V, 00 ns for ion gel and 100 µs for PIL) of devices operated as ECRAMs. Reproduced with permission [66]. Copyright 2021, Wiley–VCH

Another effective approach is to incorporate ion-trapping or ion-blocking materials for manipulating the ion dynamics. For example, Ji et al. reported ion-trapping concept through utilization of a PEDOT:Tos/PTHF composite as the active layer in achieving STP and LTP functionalities, where the crystalline PTHF serves as ionic transport barrier [34] (Fig. 10a, b). On the other hand, Burgt demonstrated synaptic features by inserting a Nafion membrane between the channel and the gate electrode to alter the dynamics of ion transport across the membrane in the electrolyte (Fig. 10c) [51,133]. An OECTs without a membrane exhibits a faster response speed than one with a membrane. However, the presence of a membrane, facilitating ion diffusion, results in longer ion retention times in the channel and higher residual channel currents. Zhang et al. also highlighted the use of triethylene glycol (TEG) chains in coordinating cations, effectively slowing their removal from the film. This interaction is critically dependent on the density of TEG chains, where a higher density correlates with reduced exfiltration rates. Consequently, polymers substituted with double TEG chains have demonstrated exceptional conductance state retention, surpassing two orders of magnitude, in ECRAM devices [134].

In a seminal study by Melianas et al. introduced the use of solid electrolytes formed by infiltrating an electrically insulating polymer with common ionic liquids. This development enables the programming of organic ECRAMs in a vacuum using low voltage (± 1 V) and sub-microsecond pulses, with fast-switching capabilities reaching down to 20 ns. A particularly interesting observation in fast potentiation and depression measurements is the spike-and-recovery feature exhibited by the *I*_DS_ (*G*_SD_) during the “read” pulse (Fig. 10d). This feature, which significantly constrains the system’s ability to achieve a steady state, represents a common phenomenon across various ECRAM systems, yet its underlying mechanisms remain poorly understood. This behavior is related to the “write” process, specifically during periods when the *V*_SD_ is set to zero, resulting in uniform doping of the channel since *V*_GS_ equals *V*_GD_, and excessive doping when *V*_GS_ exceeds the later applied V_SD_. This excessive and uniform ion distribution leads to an instantaneous surge in *I*_DS_ due to increased charge carriers and the absence of a diffusion current [20].

Ion trapping and blocking and applying gate voltage to enable the ion transport are usually combined to achieve short- and long-term potentiation [110,135]. At lower voltages, ions typically form an electrical double layer (EDL) at the channel interface, leading to rapid ion drift-back post-voltage switch, accounting for short-term potentiation. Higher gate voltages cause ions to penetrate and partially remain in the channel, resulting in quasi-permanent conductance changes indicative of long-term potentiation. Successive low voltage pulses can also convert short-term potentiation into long-term potentiation. For example, artificial synapses utilizing P3HT–PEO core–sheath organic nanowires have been shown to exhibit synaptic responses [128]. Initially, ions ([TFSI^−^] anions and [EMIM^+^] cations) are randomly distributed within the ion gel. Upon applying a negative presynaptic spike, anions accumulate near the channel, forming an EDL and thereby generating holes in the channel. Additionally, some anions penetrate the PEO:P3HT, inducing more charge carriers within the channel. Once the presynaptic spike ceases, these ions gradually return to the ion gel medium. The retraction of these anions decreases the induced charge carriers in the channel, leading to a synaptic-decay response.

The electrolyte’s role is pivotal not only in enhancing device transient speed, but also ensuring the non-volatility of OECTs. On the one hand, different ions have different transport dynamics in the channel and ion-blocking layers. On the other hand, the integration of anion–cation pairs affect anionic and cationic hole compensation, allows mobile ions to offset electronic charges in the semiconductor, which accelerates device response times in ion gel devices but reduces state retention due to the lowered energetic barrier for the back-diffusion of ions. Quill et al. have demonstrated that devices utilizing a polymerized ionic liquid (PIL) electrolyte exhibit significant hysteresis compared to ion gel devices, despite the use of considerably slower scan rates to offset the PIL’s reduced ionic conductivity [66] (Fig. 10e-g). This hysteresis indicates a decrease in device speed, as ion motion struggles to match the gate voltage sweep. However, the modulation capability of PIL-based devices is akin to that of ion gel-gated devices, confirming that ion penetration does occur in PIL devices to modulate the semiconductor volume, albeit at a reduced rate.

**Fig. 11:**
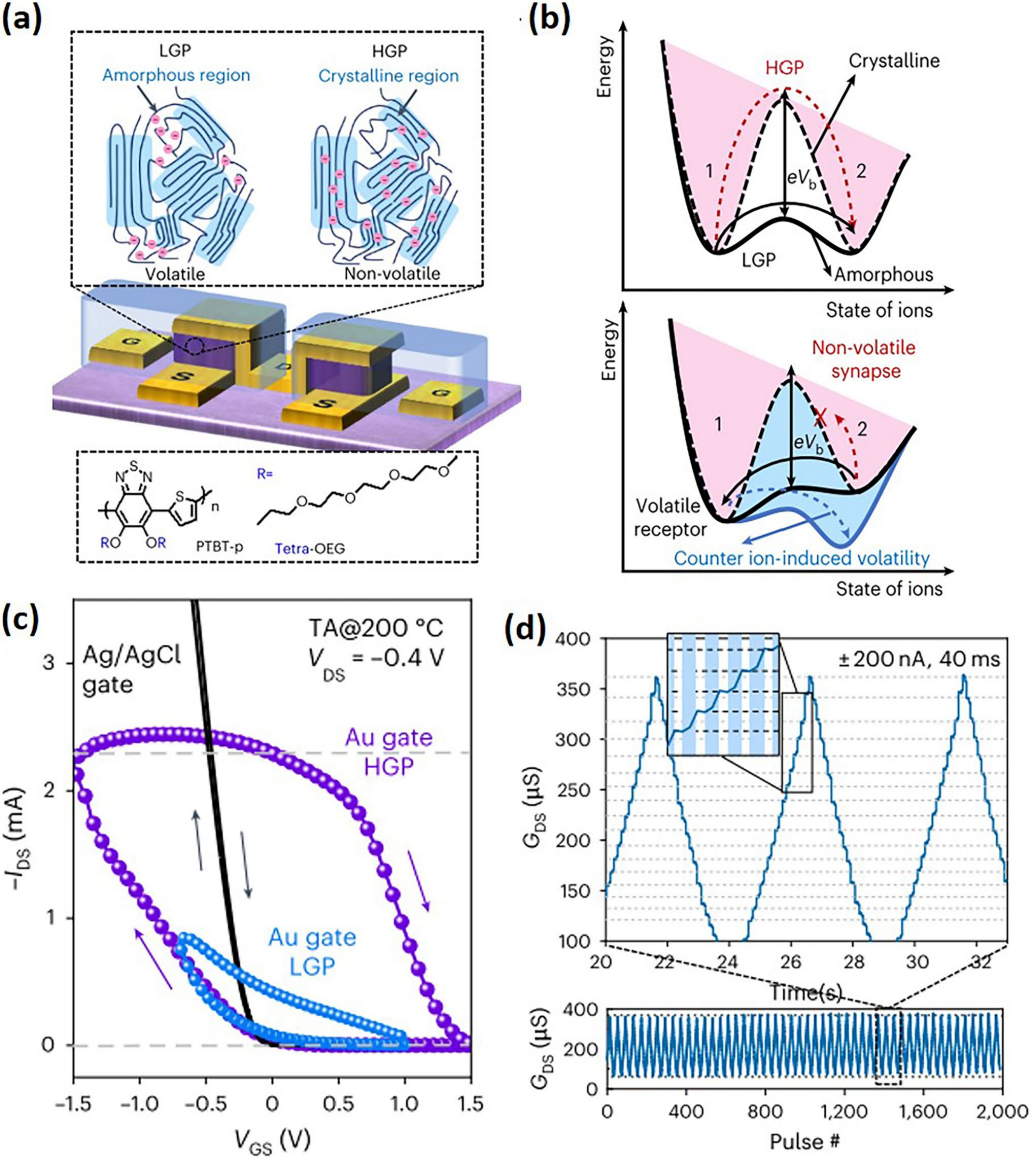
**a** Device architecture of v-OECT; the two dashed boxes show the ion contribution in the volatile/non-volatile mode and the chemical structure of PTBT-p, respectively. **b** Schematic explaining the mode-switching mechanism. The special channel dimensions and crystallization provide a high-barrier e*V*_b_ between the two ionic states (1 and 2), resulting in a non-volatile behavior. *V*_b_ denotes the voltage bias that drives the ions to overcome the barrier. **c** Transfer curves of v-OECT with polarizable/non-polarizable gate electrode. **d** Cyclic LTP under current control (2,000 pulses, ± 200 nA, 40 ms). Three reproducible LTPs with linear, symmetrical programming and one-to-one correspondence are shown (top). Reproduced with permission [4]. Copyright 2023, Nature Portfolio

The microstructure, particularly the crystallinity, also significantly affects the ionic dynamics. Enhanced crystallinity, achieved through methods like annealing, can effectively trap ions within the film’s ordered and compact side chains, only releasing them upon the application of sufficient potential, thereby ensuring device non-volatility (Fig. 11a, b). The versatility in the chemical and morphological design of organic semiconductors allows for the fine-tuning of ion injection and release rates, optimizing the write-read speeds for OECTs applications. While *p*-type OMIECs demonstrate such non-volatility, n-type OECTs face challenges due to retention performance deterioration by ORR. A proposed solution involves utilizing vertical device architectures with channels encapsulated by solid electrolytes to prevent air penetration, addressing the issue of retention performance in *n*-type OECTs [9].

Surprisingly, the structural design of OECTs also strongly affects the ion dynamics. A shift from planar to vertical structures has been observed to enhance non-volatility [4,136]. Wang et al. explored the concept of ion trapping in crystalline domains of electrochemical transistors, demonstrating the feasibility of a vertical OECTs capable of both volatile and non-volatile operations (Fig. 11). They suggest that in a vertical *p*-OECTs with an ultrathick channel (approximately 2 μm), a higher concentration of TFSI^−^ ions will maintain within the bulk of the channel due to the limited electrolyte-channel interface, leading to minimal anion migration back to the electrolyte even if the gate voltage is removed, thereby establishing a narrow neutral interface while the majority of anions are either retained internally or obstructed by large crystallites. Therefore, vertical structures inherently lead to effective ion blocking compared to planar structures.

In addition to the vertical structure, Wang et al. also demonstrated the effect of gate electrode on OECT volatility [4]. The incorporation of a non-polarizable gold (Au) electrode reveals an energy barrier of approximately 0.8 eV for embedding TFSI^−^ ions into the crystalline glycol side chains, as determined by the breakpoint potential of the sample annealed at 200 °C. Application of a sufficient gate voltage allows electrolyte ions to infiltrate the crystalline regions of the OMIEC channel, trapping them until a reverse voltage is applied, enabling the achievement of over 1,024 non-volatile analog states (10-bit). Conversely, at lower gate voltages, ion penetration is limited to the amorphous regions, resulting in volatile behavior akin to traditional transistors due to weaker interaction forces. On the other hand, they show that when employing a non-polarizable Ag/AgCl gate electrode, only volatile behavior is manifested. Despite TFSI^−^ ions remain trapped within the channel, volatility is due to the attraction between the counterions ([EMIM^+^][TFSI^−^]) and the trapped anions within the channel, leading to their compensation. This is because [EMIM^+^] ions can be reversibly reduced to a neutral state on the Au gate electrode upon the application of a negative voltage.

## Other Types of Transient Responses in OECTs

OECTs have been shown to respond to various stimuli, including electrical signals [17,24], chemical and biochemical agents [83,127,137–142], temperature variations [4], pressure sensing [143–145], and light exposure [4,146,147]. They can achieve high sensitivity or mimic artificial synaptic functions by combining OMIECs with materials that naturally respond to these stimuli or by modifying the device’s configuration [4,5,148]. This section provides a concise review of the mechanisms behind the photoresponse, chemical response, and pressure response in OECTs, and we also discuss the crucial physical processes that determine their transient responses.

### Photoresponse

Photoresponse in OECT typically adheres to one of two established paradigms. The first involves integrating photoactive materials on the gate electrode, which undergo photoelectrochemical reactions under light exposure to modulate the channel doping state and, consequently, alter the channel conductivity. The second approach capitalizes on the inherent photoactivity of OMIECs due to their conjugated backbone, allowing light to serve as an additional source—beyond gate biasing—to generate free charge carriers within the transistor, provided that the photogenerated excitons can be efficiently separated [149].

**Fig. 12:**
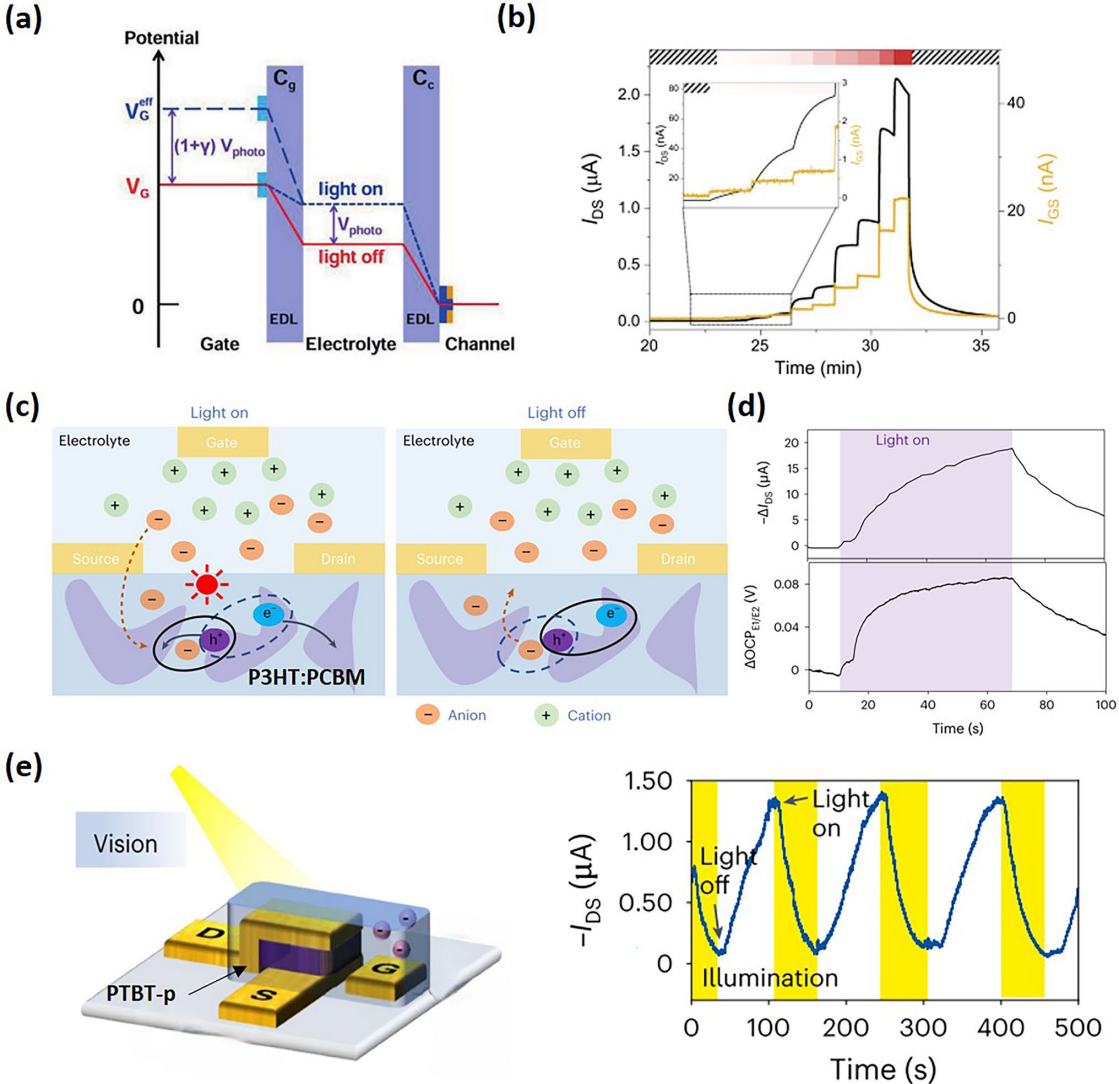
**a** Modulation of gate voltage applied on an OPECT due to the photovoltage V_photo_ induced by the light irradiation. Reproduced with permission [152]. Copyright 2018, Wiley–VCH. **b** Current–time profile of n-OPECT upon increasing the light intensity from 0 to 406 mW cm^−2^. Reproduced with permission [147]. Copyright 2023, Druet et al. **c** Schematic illustrations of photon-modulated electrochemical doping. Light-induced charge carriers in the bulk heterojunctions lead to ion transport from the electrolyte for charge compensation. After the light illumination, the presence of anions prohibits the immediate charge recombination. **d** Simultaneous channel current change and open-circuit potential (OCP_E1/E2_) change in response to light illumination. (**c, d**) Reproduced with permission [146]. Copyright 2023, Nature Portfolio. **e** Structure diagram of v-OECT and its non-volatile response to light signal. Reproduced with permission [4]. Copyright 2023, Nature Portfolio

Most reported OECTs with photoresponse belong to the first category, utilizing a Faradaic photo detection mechanism where photogenerated electrons, extracted at the gate electrode, facilitate the modulation of the channel current [150–152]. The operational mechanism of such OECTs is illustrated in Fig. 12a, b. Given the conductive nature of the electrolyte, the gate voltage effectively applies across two interfaces within the OECTs: the gate/electrolyte and electrolyte/channel (organic semiconductor) interfaces. Each interface hosts an electric double layer (EDL) that functions similarly to a capacitor. Thus, the gate and channel EDLs, with capacitances *C*_gate_ and *C*_channel_ respectively, are connected in series within the device. In the absence of light illumination, the potential drop at the electrolyte/channel interface, enabling a shift of the transfer curve to a lower gate voltage under light irradiation [152]. The exciton separation (~ ps) and charge transfer are fast [147]; however, ion doping process is usually much slower, same as the gate voltage modulation. As such, the response time is determined by the speed of the photosensitizer or OECTs, primarily influenced by the rate of ion doping.

Another category of OECTs photoresponse devices involves utilizing the OMIEC material as a photoactive layer. For instance, Chen et al. utilized a P3HT:PCBM bulk heterojunction as the channel for the OECTs to emulate ion flux-modulated synaptic activity and construct an optically controlled optoelectronic synapse (Fig. 12c, d). They demonstrated that light absorption can perturb electrochemical doping, and the light-induced charge carriers in the bulk heterojunction leads to ion transport from the electrolyte to the channel for charge compensation. In addition, light acts as a presynaptic input to generate a postsynaptic electrical signal. The resultant photon-induced ion diffusion facilitates synaptic behaviors and memory effects. It should be noted that in this scenario, the device exhibits significant photocurrent at low operating voltages (< 1 V), enabling high-density, non-volatile electrical conductance states for neuromorphic computing. When the light is turned off, the presence of anions around the doped P3HT inhibits immediate charge recombination, leading to a slow current decay, which contributes to the formation of non-volatile memory currents.

The modulation is influenced by both light intensity and gate voltage, whereas the memory effect is primarily governed by the gate voltage that modulates ions for different morphology phases. In such devices, exciton generation and separation occur rapidly (< ns); hence, the transient response speed during the device’s on and off states is governed by ion transport, similar to electrical modulation. Wang further demonstrated the use of a single-component PTBT-p and EMIMTFSI as the channel and electrolyte, respectively, in a vertical OECTs that exhibits good electrical non-volatility (Fig. 12e). Under light illumination, the *I*_DS_ immediately increases, but the response time is significantly slower than that of electrical pulse modulations. The physics mechanism for this sustained *I*_DS_ increment—or why the light response time is so slow compared to electrical modulation—in such a device structure remains unclear. After turning off the light, the *I*_DS_ shows a very slow decay rate, akin to electrical non-volatile performance, acting as a non-volatile light response [4,146].

### Chemical and Biochemical Response

OECTs have gained tremendous attraction in chemical and biochemical sensings including ion concentration sensing [8,138,153], glucose sensing [83,142], antigen–antibody sensing [154,155], DNA sensing [139,140,156], bacteria sensing [141] and virus sensing [137], due to their outstanding performance with high sensitivity, flexibility, selectivity, real-time monitoring, ease of fabrication, and biocompatibility [157]. This high level of sensitivity holds significant value for medical diagnosis, environmental monitoring, and food safety inspection. Understanding the mechanisms behind OECT’s response to molecular interactions can help us further improve response times, achieving quicker detection of various molecules.

**Fig. 13:**
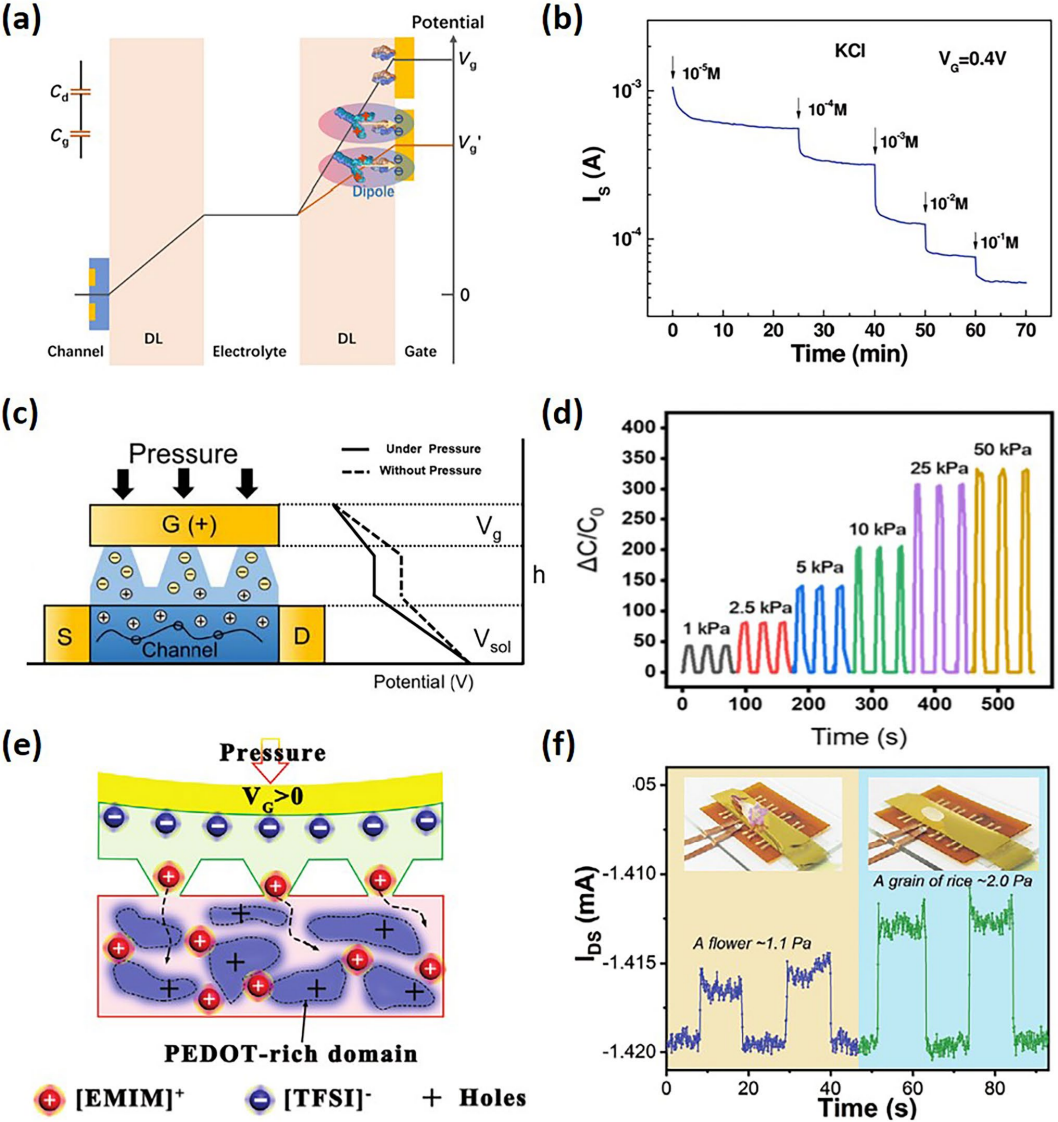
**a** Schematic diagram of the operation principle for protein sensing. The arrow in the dipole shows the electric field generated by the positive charge of protein on the gate surface. Reproduced with permission [137]. Copyright 2021, American Association for the Advancement of Science. **b** Current response to ion concentration in electrolyte. Reproduced with permission [158]. Copyright 2010, American Chemical Society. **c** Schematic diagram of the microstructured hydrogel-gated OECT pressure sensor. Under pressure, the change in gate/microstructure area increases the effective gate voltage across channel. **d** Real-time change of *I*_DS_ to pressure. Reproduced with permission [162]. Copyright 2020, American Chemical Society.** e** Schematic illustration of the contact-modulated ionic doping mechanism. **f** Real-time change of *I*_DS_ to pressure. Reproduced with permission [145]. Copyright 2020, Wiley–VCH

The first mechanism involves the response of OECT’s channel to ion concentration within electrolytes [8,153]. Lin et al. reported the Nernstian relationship between gate voltage shift and cation concentration (Fig. 13a) [158]. As the concentration of cations in the electrolyte increases, the transfer curve of the OECTs shifts toward lower gate voltages. The response speed, influenced by the diffusion rate of ions and volume of electrolytes, completing in few minutes, through considering simple estimation of $t=\frac{{x}^{2}}{2D}$, where *x* is the diffusion distance and *D* is the ion diffusion coefficient (Fig. 13b).

The second mechanism, usually applied for biochemical sensings, achieves specificity by modifying the gate electrode of the OECTs, enabling it to specifically bind with certain molecules, thereby affecting the channel current through changes in surface charge at the gate [138,159–161]. For instance, by modifying the gold gate electrode with a self-assembled monolayer (SAM) and SARS-CoV-2 spike protein, the binding of IgG molecules to the spike protein on the gate surface forms electric dipoles, altering the gate surface’s potential and causing changes in the OECTs channel current. Experimental results demonstrate that the detection time for IgG can be reduced to 2 min using voltage pulses, although the electrical response in non-modified device is much faster [159]. Applying the alternating current electrothermal flow (ACET) has been shown to substantially concentrate the target molecules to the immobilized nanobodies, enable the fast chemical molecular detection within 2 mins [155].

### Pressure Response

The gate-electrolyte capacitance (*C*_ge_) is small (Fig. 13c, d) [144]. The other mechanism involves changing the contact area between the solid electrolyte and the channel to modulate the local conductivity of the channel, thus altering the effective conductance of the entire channel [145,162]. This do not require the use of a non-Faradic electrode, avoiding additional capacitance. Additionally, it is noteworthy that devices employing depletion mode PEDOT:PSSbility of OECTs to respond to pressure signals holds promising application prospects in the field of tactile sensing. Pressure sensors based on OECTs often design the electrolyte that transports and stores ions into a special pressure-sensitive structure, with micro-pyramid arrays being a common type of electrolyte structure [62,144,145]. The pressure sensing mechanism of OECTs can be categorized into two types. One mechanism operates by altering the non-Faradic gate electrode and solid electrolyte area through pressure, regulating the relative capacitance ratio between the gate/electrolyte and channel. This change affects the effective gate voltage that modulates the channel and thereby alters the conductance. Such a structure utilizes the non-Faradic electrode, adding additional capacitance to the ionic circuit, leading to a slowdown in ion doping, particularly the condition of under minor pressure, when OECTs, which have a higher charge mobility, exhibit faster electrical response speeds compared to the first type (Fig. 13e, f). Interestingly, Zhang et al. reported a pressure sensor based on this mechanism with synaptic functions, demonstrating both short-term and long-term memory in response to pressure signals [143]. The synaptic characteristics primarily involve increased pressure enhancing the contact area between the solid electrolyte and the channel, which allows more cations to enter under gate voltage and remain there even after the pressure is removed, due to the minimal contact at the tip of the electrolyte pyramid that blocks the return of ions. However, in current OECTs pressure sensors, the pressure response time (in seconds) is still slower than the corresponding electrical response time (in milliseconds) [145]. This could be attributed to a variety of reasons: Firstly, the rate at which the electrolyte is strained; secondly, the reduced mobility of ions within solid electrolytes; and thirdly, the constraints on microstructure size result in pressure-sensing OECTs having larger channel sizes, which in turn causes a decrease in the speed of electrical responses.

## Conclusion and Outlook

The recent rapid advancement in OECTs has led to the introduction of new devices exhibiting superior performance across various applications, including logic transistors, intelligent sensing, artificial synapses, ECRAM, and more. Significant experimental progress has been achieved, continually enhancing the performance of OECTs. A deep understanding of the device’s operational mechanisms and the fundamental principles of ion–electron transduction is crucial for quick and effective device optimization. The unique dynamics of ion transport are particularly critical for the transient response of OECTs. For applications such as logic transistors and intelligent sensing, rapid ion transport is desirable, aiming to reach or even surpass the speeds of OFETs and silicon transistors. Conversely, for applications requiring non-volatility, like artificial synapses and ECRAM, the ion dynamics of the back-diffusion process is different from the volatile type. This means being relatively quick to replicate STP functions, slow for LTP, and potentially eliminating back-diffusion for ECRAM applications. This review provides a comprehensive examination of device physics and highlights several outstanding works on modeling OECTs, especially focusing on the transient response behaviors of OECTs. Considering the highly multidisciplinary nature of the OECTs community, our primary goal was to elucidate the unique device characteristics and models of OECTs arising from their mobile ion properties, and then to discuss how these mobile ions and electrochemical reaction principles influence the transient response. Additionally, we explored the mechanisms and strategies for controlling slow ion dynamics in non-volatile OECTs. Furthermore, we broadened the scope of OECTs transient responses to include photo-response, pressure response, and molecular sensing, demonstrating that these processes are intimately linked to the intrinsic ion dynamics within the OECTs.

Despite significant advancements in OECTs research, several challenges and difficulties remain. The first challenge is the contradictory relationship between *g*_m_ and time constant *τ* commonly suffered in OECTs, where a higher *g*_m_ necessitates increased charge carrier mobility, which, however, adversely affects ion transport. A high volumetric capacitance C^*^ also seems to slow down the ionic circuit charging process. Therefore, future efforts should focus on material optimization, innovative morphology control, and device structure enhancement to improve both gm and the speed of transient response. The second challenge relates to integration issues. As the OECTs device area decreases (for either faster response speed or higher array integration), the relative redundant area compared to the channel increases. This can include the extrachannel OMIEC film and the electrode area. Since OMIEC is ion-conductive, ions can penetrate through the channel and come into contact with the source/drain electrodes (typically Au), leading to additional capacitive effects. The smaller the device, the more pronounced such effects will be. Furthermore, device-to-device non-uniformity, including variations in *g*_m_, *τ*, *V*_th_, etc., poses a significant issue for integration, requiring future attention. Third, the theoretical mechanisms underlying the effects of crystallinity, morphology, doping depth, device structure, and ion transport activation energy on slow ion dynamics—critical for mimicking STP, LTP, and even ECRAM—are still lacking. Current approaches are largely empirical, but deeper theoretical investigations in the future could enable more precise control over OECTs non-volatility behaviors, achieving higher performance. Fourth, based on the third point, the switching speed and retention of states in ECRAM become especially crucial. Research indicates that ECRAM can support more states than other devices like ReRAM and PCM, yet it does not have an advantage in state retention, and its write speed is a significant drawback. Future research aimed at improving the write speed of OECTs-based ECRAM will be an important direction. Considering our limitations, there may be other relevant future directions we have not emphasized. Nonetheless, we hope this review highlights the fascinating device physics of ion dynamics and transient response in OECTs, encouraging more scientists to contribute toward advancing superior OECTs performance across various application scenarios.
